# Reproducible chiroptical activity from aggregated chiral thienopyrroledione–fluorene π‑conjugated polymers

**DOI:** 10.1080/14686996.2026.2680968

**Published:** 2026-06-02

**Authors:** Nao Suzuki, Ziwei Hu, Sota Nakayama, Soh Kushida, Yohei Yamamoto, Wijak Yospanya, Reiko Oda, Takaki Kanbara, Junpei Kuwabara

**Affiliations:** a Institute of Pure and Applied Sciences, University of Tsukuba, Tsukuba, Ibaraki, Japan; b Tsukuba Research Center for Energy Materials Science (TREMS), Institute of Pure and Applied Sciences, University of Tsukuba, Tsukuba, Ibaraki, Japan; c Advanced Institute for Materials Research (AIMR), Tohoku University, Sendai, Miyagi, Japan; d CNRS, Bordeaux INP, CBMN, UMR 5248, University of Bordeaux, Pessac, France

**Keywords:** Chiral π‑conjugated polymer, thienopyrroledione, circular dichroism, circularly polarized luminescence

## Abstract

Chiral π‑conjugated polymers have attracted considerable interest because their chiral-aggregated structures can generate pronounced chiroptical responses. Here, we report the design, synthesis, and chiroptical characterization of fluorene – thienopyrroledione polymers bearing a single chiral center in each repeating unit, **(*R*)-** and **(*S*)-PFTPD**, prepared *via* direct arylation polycondensation to minimize structural defects. Although only weak chiroptical activity was observed in the solution, the addition of 1‑butanol to CHCl_3_ solutions of the polymers induced the formation of stable aggregates that exhibited strong circular dichroism (CD) and circularly polarized luminescence (CPL). Systematic examination of the sample preparation parameters revealed that the mixing process critically influences the chiroptical properties. Dropwise addition of 1-butanol under controlled stirring provided the most reliable results, yielding highly reproducible CD spectra. Under these optimized conditions, the aggregates exhibited an average dissymmetry factor (|*g*_abs_|) of 1.5 × 10^−2^ and a CPL dissymmetry factor (|*g*_lum_|) of 1.9 × 10^−2^. These values rank among the highest reported for π-conjugated polymer aggregates bearing only a single chiral source per repetition unit. This study demonstrates that precise control over the solvent-mixing process is essential for the reproducible emergence of chiroptical properties and highlights the chiral TPD unit as a promising platform for chiroptical polymer materials.

## IMPACT STATEMENT

This study demonstrates highly reproducible, strong CD and CPL from chiral π-conjugated polymers, enabled by a robust and standardized aggregate-formation protocol.

## GRAPHICAL ABSTRACT

**Figure:**
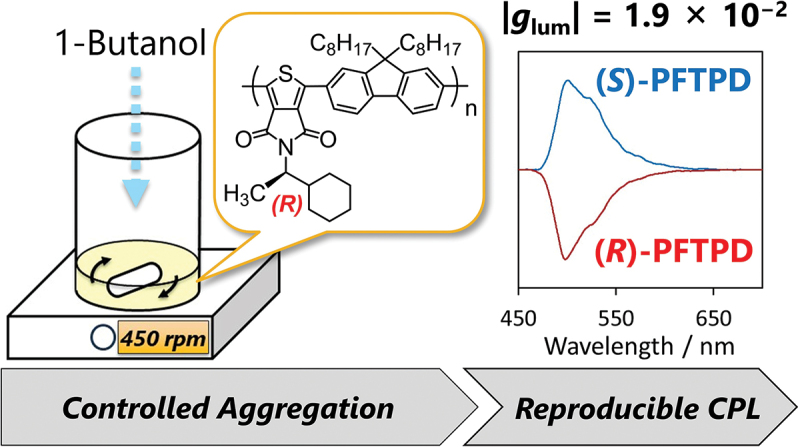

## Introduction

**Figure 1.**
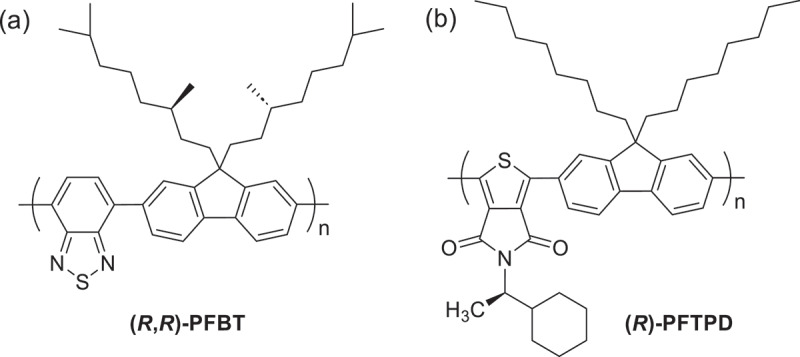
(a) Conventional fluorene-based chiral polymer **(*R,R*)-PFBT** and (b) the target polymer bearing a chiral TPD unit developed in this work **(*R*)-PFTPD**.

Chirality is a fundamental structural motif that manifests across multiple length scales from molecular to supramolecular and even macroscopic dimensions, and plays a key role in determining the properties of functional organic materials. In π-conjugated systems, the combination of extended electronic delocalization and chiral organization offers unique opportunities for controlling light – matter interactions, enabling phenomena such as circular dichroism (CD) and circularly polarized luminescence (CPL) [1,2]. The chiroptical properties of π-conjugated materials are governed not only by their intrinsic molecular asymmetry but also, and often predominantly, by their supramolecular organization in thin films or aggregated states [3–5]. Chiral side-chain – functionalized π-conjugated polymers such as poly(*p*-phenylene)s [6–8], poly(*p*-phenylene-vinylene)s [9], and polythiophenes [10,11] have long been studied for their chiroptical properties. In particular, π-conjugated polymers containing fluorene units exhibit distinctive optical activity arising from their unique aggregation behavior and liquid-crystalline ordering [12–19]. The fluorene unit’s rigid backbone and strong propensity for self-organization, combined with precise control over intermolecular packing through 9,9-disubstitution (Figure 1a), underlie these distinctive chiroptical properties. Introduction of chiral side chains onto fluorene units has been recognized as a key strategy for generating pronounced CD and CPL responses [17]. Nevertheless, the reliance on conventional chiral building blocks limits the accessible structural diversity. In contrast, the thienopyrroledione (TPD) unit offers a highly versatile platform for molecular design (Figure 1b) [20]. Because its substituent can be directly derived from primary amines, a wide variety of readily available chiral amines can be seamlessly incorporated as chiral side chains. This straightforward and modular approach greatly expands the accessible library of chiral monomers, enabling systematic tuning of molecular asymmetry and facilitating the creation of structurally diverse chiral conjugated polymers. Moreover, TPD is a strong electron-accepting unit known to impart high chemical stability, distinctive aggregation behavior, and suitability for use in organic semiconducting polymers [20,21]. For an accurate understanding of the intrinsic chiroptical properties of chiral polymers, it is also crucial to prepare polymers with minimal structural defects along the main chain. We have previously demonstrated that π-conjugated polymers containing TPD can be synthesized *via* direct C – H arylation polycondensation [22–25], enabling precise control over backbone structure and chain ends [26,27]. When properly optimized, this method affords defect-free polymers more efficiently than conventional cross-coupling polymerizations and is therefore highly suitable for fundamental studies on chiral conjugated systems. In this work, we designed and synthesized chiral TPD monomers derived from chiral amines and prepared copolymers with achiral dioctyl-substituted fluorene units *via* direct arylation polycondensation. By systematically investigating their CD and CPL responses, we aimed to elucidate the role of the chiral TPD unit in governing the chiroptical properties of the resulting polymers. During this investigation, we encountered significant issues in the reproducibility of the chiroptical responses. To overcome this issue, we established an experimental protocol that enables highly reproducible observation of CD and CPL responses from the polymers. Notably, this protocol directly addresses a critical challenge common to aggregated and solid-state chiral π-conjugated systems, namely the poor reproducibility of chiroptical responses arising from subtle differences in sample preparation and processing conditions [3,9,28–30]. Despite containing only a single chiral source within each repeating unit, the polymers exhibited CPL dissymmetry factors on the order of 10^−2^ in the aggregated state. These values are an order of magnitude larger than those typically reported for polymer aggregates dispersed in solution (∼10^−3^). These findings demonstrate that the chiral TPD unit is highly effective in inducing supramolecular chirality and provides a new molecular-design principle for chiral π-conjugated polymers.

## Results and discussion

### Synthesis and characterization of polymers

**Figure 2.**
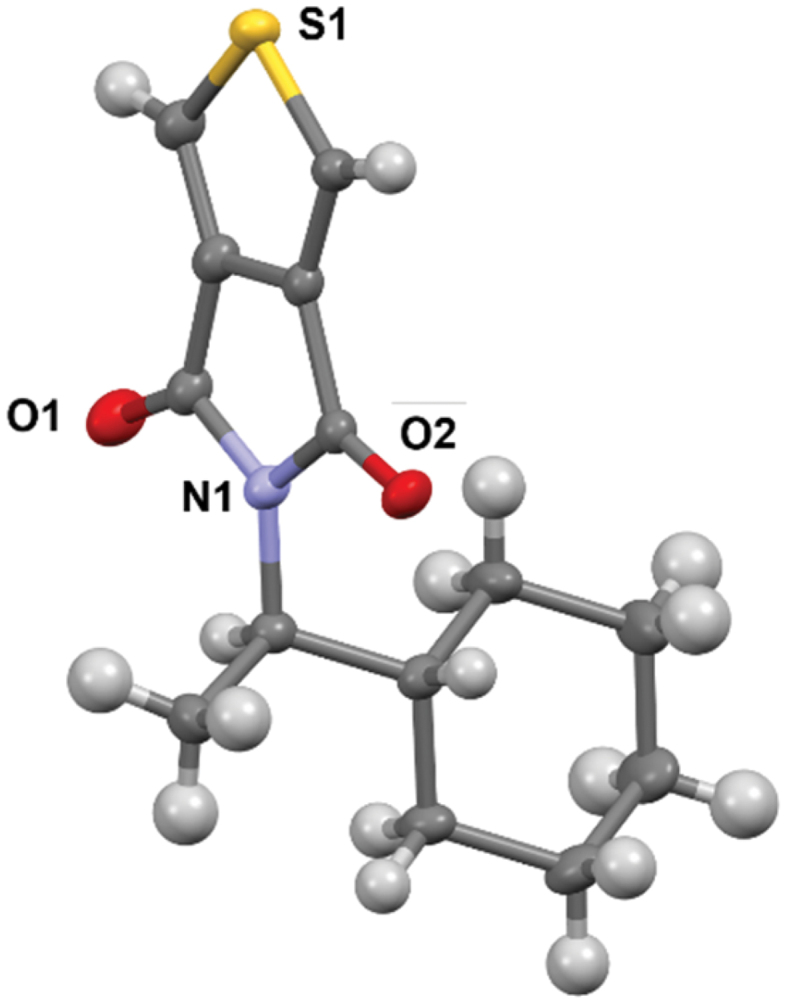
Crystal structure of **(*R*)-TPD** with thermal ellipsoids shown at the 50% probability level.

**Scheme 1.**
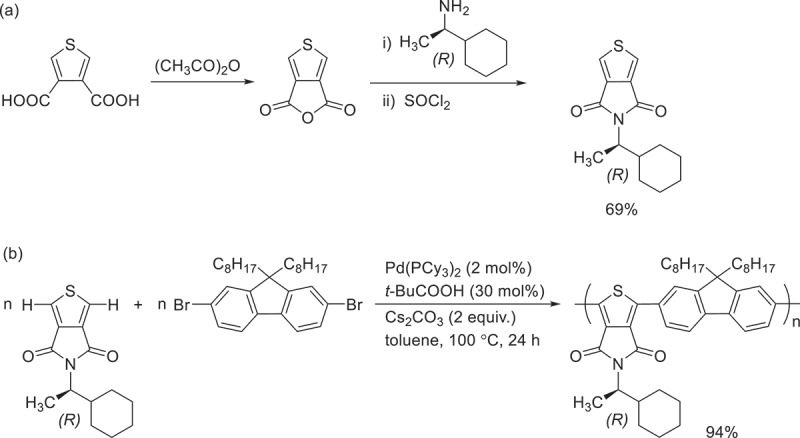
(a) Synthesis of **(*R*)-TPD**; (b) synthesis of **(*R*)-PFTPD** *via* direct arylation polycondensation.

In this study, (*R*)-1-cyclohexylethylamine was selected from among readily accessible chiral amines. This choice was motivated by the fact that the chiral center is positioned directly adjacent to the nitrogen atom, allowing it to be placed in close proximity to the polymer backbone when incorporated into the TPD unit. Such proximity is expected to enable the TPD side chain to more effectively influence the chiral organization of the polymer through intermolecular π–π stacking upon aggregation. In addition, the cyclohexyl group ensures sufficient solubility of the polymer. The TPD bearing the (*R*)-1-cyclohexylethyl side chain, **(*R*)-TPD**, was synthesized following the synthetic procedure reported for the corresponding achiral side-chain derivative (Scheme 1a) [31]. After converting 3,4-thiophenedicarboxylic acid to the corresponding anhydride, the intermediate was reacted with (*R*)-1-cyclohexylethylamine without isolation, followed by dehydration to afford the desired product with an overall yield of 69%. The obtained compound was characterized by ^1^H and ^13^C NMR spectroscopy, mass spectrometry, and elemental analysis. Using ^1^H-^1^H COSY NMR in combination with a DFT-calculated chemical-shift prediction, all signals observed in the^1^ H NMR spectrum were fully assigned (Figure S1-S3). The three-dimensional structure was ultimately elucidated by single-crystal X-ray diffraction analysis (Figure 2). Two crystallographically non-equivalent molecules were present in the unit cell, and one of the cyclohexyl groups exhibited disorder. Figure 2 shows the structure without the disordered component. Both the cyclohexyl and methyl groups adopt orientations above and below the TPD plane, respectively, presumably to minimize steric repulsion.

When the direct arylation polycondensation between **(*R*)-TPD** and 9,9‑dioctyldibromofluorene was conducted under the optimized conditions (Scheme 1b) [27], **(*R*)-PFTPD** was obtained in 94% yield with an *M*_n_ of 45,000 (Table 1). The structure of the obtained polymer was confirmed by^1^ H NMR spectroscopy and matrix‑assisted laser desorption/ionization time‑of‑flight mass spectrometry (MALDI‑TOF MS) (Figures S4 and S5). The MALDI‑TOF‑MS spectrum of **(*R*)-PFTPD** indicates the absence of structural defects, such as homocoupling, and show that most chain termini, are capped with the TPD unit. The presence of the TPD terminal group is also confirmed by the^1^ H NMR spectrum (Figure S4).

**Table 1.** Results of polycondensation reactions and photophysical properties of the polymers in solution.

Polymer	Yield / %	*M*_n_^a^	*M*_w_/*M*_n_^*a*^	λ_abs_^b^ / nm	λ_em_^c^ / nm	Φ^d^ / %
*(R)*-PFTPD	94	45,000	2.1	463	487	53
*(S)*-PFTPD	92	56,000	2.2	463	487	53

^*a*^Estimated by GPC calibrated on polystyrene standards, using CHCl_3_ as an eluent at 40°C. ^*b*^Absorption maximum wavelength in CHCl_3_ solution, ^*c*^Emission maximum wavelength in CHCl_3_ solution, ^*d*^Photoluminescence quantum yield.

The enantiomeric **(*S*)-TPD** and **(*S*)-PFTPD** were synthesized in the same manner. The obtained monomers and polymers exhibited nearly identical NMR and MALDI‑TOF MS spectra (Figure S6-S8). In addition, **(*R*)-PFTPD** and **(*S*)-PFTPD** exhibit comparable molecular weights (Table 1). These results allow direct comparison of their chiral properties without contributions from differences in structural defects or molecular weights. The polymer was confirmed to be amorphous, as evidenced by the absence of distinct diffraction peaks in the X-ray diffraction (XRD) pattern and the lack of any crystallization- or melting-related thermal transitions in the differential scanning calorimetry (DSC) thermograms (Figures S9 and S10).

### Photophysical properties in solution and film states

To investigate the fundamental physical properties of the polymers, absorption, and photoluminescence spectra were measured in both the solution and thin-film states (Figure S11). In solution, the polymer showed a maximum absorption at 463 nm and an emission maximum at 487 nm (Table 1). Time-dependent density functional theory (TD-DFT) calculations indicate that the longest-wavelength absorption band originates from a π–π transition along the polymer backbone (Figure S12). The absorption and emission spectra of the spin-coated thin films were slightly red-shifted (λ_abs_ = 465 nm, λ_em_ = 495 nm) relative to those observed in solution, suggesting that solid‑state packing causes only slight modifications to the electronic states (Figure S11b).

### Chiroptical properties and reproducibility

**Figure 3.**
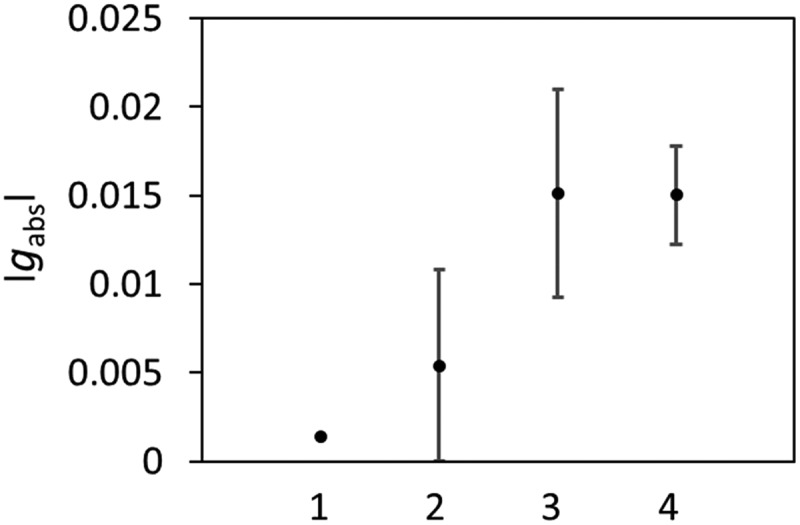
Dependence of the |*g*_abs_| values on the sample preparation conditions. Error bars represent the standard deviations obtained from repeated measurements. Details of conditions are shown in Table 2.

The chiroptical properties were evaluated using circular dichroism (CD) spectroscopy. In CHCl_3_ solution, only a very weak CD signal was observed at a repeating-unit concentration of 3.0 × 10^−5^ M for **(*R*)-PFTPD**, consistent with the similarly weak CD activity observed for the corresponding chiral monomer, **(*R*)-TPD** (Figure S13). These results indicate that the individual chiral centers do not generate sufficient coupling between electric and magnetic transition dipole moments to produce strong CD signals in the solution state, as commonly observed for other conjugated polymers [3,6,8,9]. To promote aggregation, 1-butanol was introduced as a poor solvent. The resulting aggregates remained stably dispersed for more than 48 hr (Figure S14). When various solvent ratios were examined, a strong CD signal was observed at a CHCl_3_/1-butanol volume ratio of 40:60. Notably, examination of the reproducibility revealed that the CD intensities varied by more than an order of magnitude depending on the sample preparation conditions (Table S1). Such variations in the CD and CPL intensities arising from differences in preparation methods or processing procedures have also been reported previously [9]. These observations underscore the importance of reproducibility in the chiroptical properties of the aggregates and motivate us to identify the key factors required to obtain consistent and reliable results. When 1-butanol was slowly added to the polymer solution in CHCl_3_, thereby allowing the two solvents to diffuse gradually, the resulting sample exhibited small |*g*_abs_| values (average: 1.4 × 10^−3^, Table 2, Condition 1). One-shot addition of 1-butanol to the polymer solution, followed by manual shaking to promote mixing of the two solvents, resulted in a high |*g*_abs_| value of 7.0 × 10^−3^; nevertheless, the reproducibility was low, as indicated by a large standard deviation (±0.0054) (Condition 2). When 1‑butanol was added dropwise to the polymer solution in CHCl_3_ under stirring, even higher |*g*_abs_| values 1.5 × 10^−2^ were obtained; however, the reproducibility remained unsatisfactory (Condition 3). By standardizing the stirring speed of the polymer solution (450 rpm), highly reproducible |*g*_abs_| values with small standard deviation (±0.0028) were obtained (Condition 4). Although both Conditions 3 and 4 employed dropwise addition under stirring, controlling the stirring speed proved essential for ensuring rapid and reproducible mixing, which substantially improved the reproducibility of the |*g*_abs_| values. Collectively, these results demonstrate that the manner in which the polymer solution in CHCl_3_ is mixed with the poor solvent 1-butanol plays a crucial role in determining the chiroptical properties of the aggregates. In particular, controlled stirring during solvent mixing (Condition 4) enables highly reproducible aggregate formation and was therefore adopted for subsequent measurements (Figure 3).

**Table 2.** Dependence of the |*g*_abs_| values on the sample preparation conditions^a^.

Condition	Mixing method	Stirring speed	|*g*_abs_|^b^
1	Slow diffusion method^d^	Not applicable	0.0014^c^
2	One‑shot addition method^e^	Not applicable	0.0070 ± 0.0054
3	Stirring method^f^	Not specified	0.015 ± 0.0059
4	Stirring method^f^	450 rpm	0.015 ± 0.0028

^a^1‑BuOH (6 mL) was added to a 75 μM solution of **(*R*)-PFTPD** or **(*S*)-PFTPD** in CHCl_3_ (4 mL).

^b^|*g*_abs_| values at the peak wavelengths around 480 nm (mean ± standard deviation).

^c^The standard deviation was not evaluated because the number of trials was insufficient.

^d^1‑Butanol was slowly poured into the polymer solution in CHCl_3_, allowing the two solvents to diffuse gradually into each other.

^e^1‑Butanol was added all at once to the polymer solution in CHCl_3_, followed by manual shaking to promote immediate mixing.

^f^1‑Butanol was added dropwise to the polymer solution in CHCl_3_ under stirring.

The pronounced dependence of the chiroptical properties on the sample preparation process suggests that the aggregation pathway plays a decisive role in defining the structure of the aggregates. In film or aggregated states, chromophores are brought into close spatial proximity, enabling intermolecular exciton coupling that strongly enhances CD responses, as widely observed for chiral π-conjugated systems [3,28]. The magnitude and shape of these exciton-coupled CD signals are therefore highly sensitive to the molecular arrangement established during aggregation [29]. In this context, Koeckelberghs and co-workers demonstrated that conjugated polymers bearing chiral side chains can follow distinct aggregation pathways depending on the rate of poor solvent addition, yielding kinetically trapped or thermodynamically stabilized structures with markedly different chiroptical properties [30]. Notably, their study showed that rapid aggregation favors a kinetically controlled, π–π-stacking-dominated structure that produces stronger CD signals, whereas slower aggregation allows relaxation into thermodynamically preferred assemblies exhibiting lower CD intensity. The behavior observed in the present **PFTPD** system is consistent with these trends. When 1‑butanol was introduced under controlled stirring to ensure rapid and homogeneous mixing, the polymers formed kinetically favored aggregates exhibiting the largest CD amplitudes (Condition 4). In contrast, slow diffusion-controlled aggregation resulted in significantly weaker CD responses (Condition 1), suggesting that the aggregates formed under such conditions adopt less cooperative chiral arrangements. The sensitivity of the CD intensity to the specific mixing protocol therefore supports the view that the strongest chiroptical responses arise from kinetically trapped aggregates, whose local packing geometry maximizes exciton coupling among **PFTPD** chromophores. Different aggregation pathways can, in principle, lead to distinct CD patterns; however, the CD spectra obtained under different mixing conditions (Conditions 1 and 4) exhibited similar spectral shapes, with variations observed only in their intensities (Figure S15). This indicates that the characteristic CD pattern originates from kinetically formed aggregates, whereas the thermodynamically favored aggregates are largely CD-inactive. Under slow aggregation conditions (Condition 1), the reduced CD intensity is therefore attributed to an increased fraction of CD-inactive aggregates. Collectively, these results reaffirm that the aggregation pathway strongly influences the chiroptical properties. Moreover, strict control over the preparation conditions is indispensable for achieving reproducible chiroptical responses. Our findings particularly underline the necessity of precisely controlling and explicitly reporting solvent addition and mixing protocols to enable reliable comparison of chiroptical data across studies.

### Effects of solvent ratio on chiroptical properties

**Figure 4.**
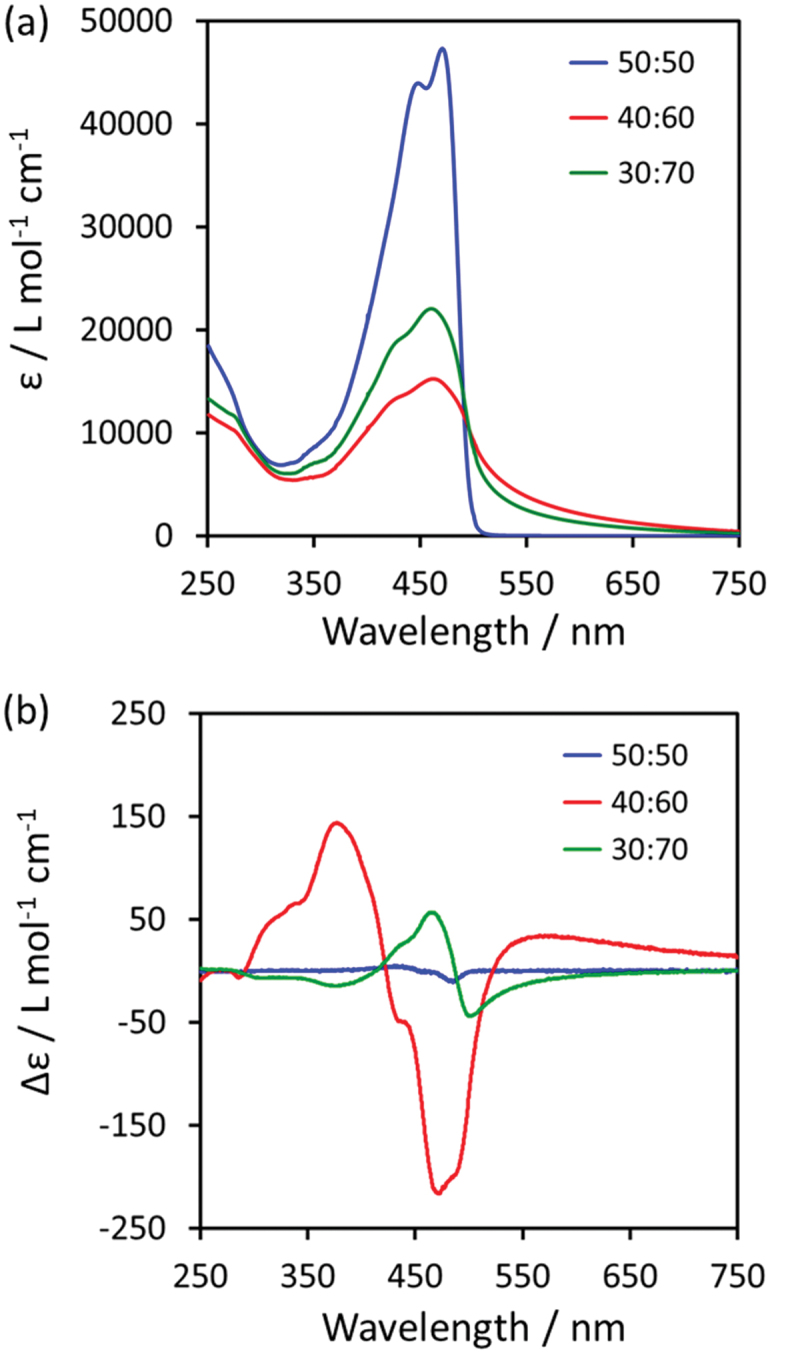
(a) UV–Vis absorption and (b) CD spectra of **(*R*)-PFTPD** recorded in CHCl_3_/1-butanol mixtures with volume ratios of 50:50 (blue), 40:60 (red), and 30:70 (green) (3.0 × 10^−5^ M).

CD spectra of **(*R*)-PFTPD** and **(*S*)-PFTPD** were measured in solvent mixtures with varying CHCl_3_/1-butanol ratios using samples prepared by an optimized solvent mixing protocol (Condition 4) to ensure reproducibility. When the volume fraction of 1-butanol was below 50%, the CD intensity remained low. Figure 4 shows the UV – Vis absorption and CD spectra obtained when the volume fraction of CHCl_3_ relative to 1‑butanol was decreased from 50:50 to 30:70. It should be noted that no precipitation was observed at any of these solvent ratios (Figure S14). At the volume ratio of CHCl_3_ to 1-butanol of 50:50, the absorption spectrum is similar to that in pure CHCl_3_, and the CD signal intensity is still low (Figure 4). These observations indicate that most of the polymer remains soluble at this solvent ratio. Upon changing the solvent ratio to 40:60, the intensity of the absorption band around 450 nm decreased, and the overall spectrum became broadened, indicating polymer aggregation. At this ratio, the aggregates exhibit a bisignated CD signal in the absorption region, accompanied by a pronounced increase in the CD intensity (Figure 4b). As the CD intensity decreases at the 30:70 ratio, the 40:60 mixture represents the solvent ratio with the highest chiroptical response. In terms of *g*_abs_, the sample in the 40:60 mixture showed the highest value, with a *g*_abs_ of −1.6 × 10^−2^ at 491 nm (Figure S16, Table S2). For all three solvent ratios examined, the CD spectra of **(*R*)-PFTPD** and **(*S*)-PFTPD** were approximately mirror images of each other (Figure S17). The observed deviation from an ideal mirror–image relationship is attributed to unavoidable technical variations in the sample preparation process.

### Characterization of aggregates

**Figure 5.**
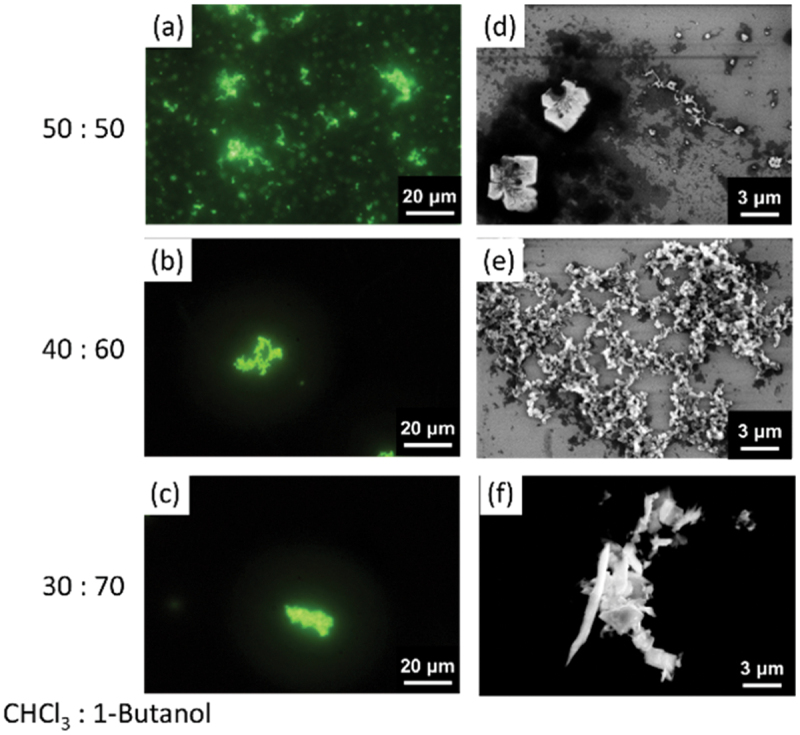
(a–c) Fluorescent microscopy images of the aggregates of **(*R*)-PFTPD** under blue – violet light irradiation in solvent mixtures of various ratios, and (d–f) SEM images of the samples prepared at each solvent ratio.

As the chiroptical properties vary depending on the solvent ratio, the corresponding aggregates were examined by optical microscopy and scanning electron microscopy (SEM) (Figure 5). When the aggregates dispersed in each solvent were observed under an optical microscope under blue – violet light irradiation, numerous fine particles exhibiting yellow-green emission were detected in a 50:50 solvent mixture (Figure 5a). In contrast, relatively large aggregates were observed in the 40:60 and 30:70 mixtures (Figure 5b,c). To examine the morphological differences in detail, SEM observations were performed. In the sample prepared from the 50:50 solvent mixture, film-like deposits were observed on the substrate (Figure 5d). Given that most polymers are dissolved in a 50% CHCl_3_ mixture solvent, it is expected these dissolved polymers precipitate to form a thin film on the substrate in a drying process. The SEM image of the sample prepared in the 40:60 mixed solvent reveals that the aggregates consist of very small, interconnected particles (Figure 5e). In contrast, the sample prepared in the 30:70 mixed solvent shows aggregates with a smooth surface morphology (Figure 5f). Because SEM measurements were carried out after solvent evaporation, the observed morphologies do not necessarily represent the aggregate structures present in dispersion. Nevertheless, qualitative differences in surface morphology were observed among samples prepared at different solvent ratios. Notably, aggregates formed at the 40:60 CHCl_3_/1-butanol ratio showed a distinct fine particulate morphology, which was not clearly observed under the other conditions.

### CPL properties

**Figure 6.**
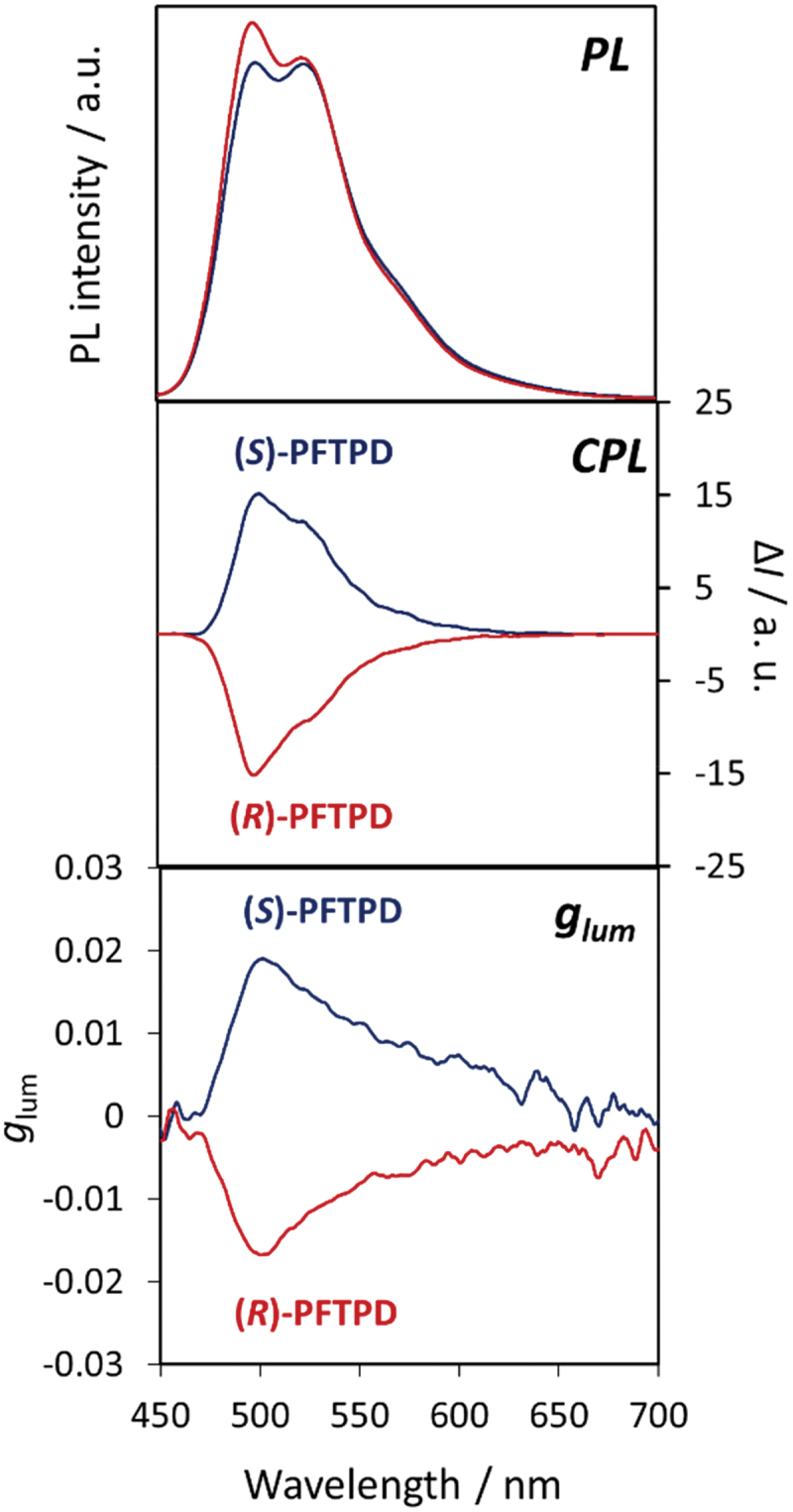
PL spectra, CPL spectra, and *g*_lum_ factors of **(*R*)-PFTPD** (red) and **(*S*)-PFTPD** (blue) recorded in CHCl_3_/1-butanol mixtures with volume ratios of 40:60 (3.0 × 10^−5^ M, λ_ex_ = 400 nm).

In a 40:60 mixed solvent of CHCl_3_ and 1-butanol, **(*R*)-** and **(*S*)-PFTPD** exhibit emission spectra with maxima at 500 nm (Figure 6). The photoluminescence quantum yield was determined to be 33%, which is relatively high for aggregates of conjugated polymers. Aggregates of **(*R*)-PFTPD**s in the mixed solvents exhibited a CPL signal with a *g*_lum_ of −1.7 × 10^−2^ at 500 nm, while **(*S*)-PFTPD** gave a mirror-image CPL signal of opposite sign. For this solvent ratio, measurements were performed on six independently prepared samples, yielding an average |*g*_lum_| value of 1.9 × 10^−2^ with a standard deviation of 4.9 × 10^−3^, demonstrating good reproducibility (Table S3). In general, conjugated polymers bearing chiral side chains typically exhibit *g*_lum_ values on the order of 10^−3^ in solution and for aggregated samples in poor solvents [32–40]. In this context, the *g*_lum_ values on the order of 10^−2^ observed in the present system are notable for solution-based polymer aggregates prepared without long-range structural ordering. Previous studies have demonstrated that exceptionally large *g*_lum_ values approaching 10^−1^ can be realized in highly ordered interchain helically π-stacked polymer assemblies, achieved by combining cationic polymers with suitably designed anionic π-conjugated molecules to precisely control π–π interactions.^7^ In contrast, the present system relies on a minimal molecular design, yet still affords *g*_lum_ values on the order of 10^− 2^, underscoring the effectiveness of the chiral TPD unit in promoting supramolecular chirality upon aggregation.

## Conclusion

In conclusion, we have shown that the thienopyrroledione (TPD) unit bearing a chiral side chain functions as an effective chiral building block for π‑conjugated polymers, enabling pronounced chiroptical activity upon aggregation. Direct arylation polycondensation afforded structurally well‑defined fluorene – TPD polymers with a single chiral center per repeat unit, allowing their intrinsic properties to be assessed without interference from structural defects. Although the CD activity in the solution was weak, mixing CHCl_3_ solutions with 1‑butanol produced stable aggregates displaying strong CD and CPL responses. Systematic evaluation of the preparation conditions revealed that the solvent mixing protocol plays a decisive role in both the emergence and reproducibility of chiroptical activity. The addition of the poor solvent under controlled stirring produced the most consistent aggregates, yielding reproducible |*g*_abs_| and |*g*_lum_| values of 1.5 × 10^−2^ and 1.9 × 10^−2^, respectively – remarkably high considering that each repeat unit contains only a single chiral center. Such preparation conditions should be precisely controlled and explicitly reported in studies of self-assembled chiroptical systems. Furthermore, the strong dependence of the CD intensity on the mixing method is consistent with a pathway in which kinetically trapped aggregates maximize exciton‑coupled chiroptical enhancement among PFTPD chromophores. This study demonstrates that the chiral TPD unit inherently possesses a high potential for inducing strong chiroptical responses, which can be fully realized through precise control of the aggregation process.

## Experimental

^1^H and ^13^C{^1^H} NMR spectra were recorded using Bruker AVANCE-400 NMR spectrometer and AVANCE-600 NMR spectrometer. Tetramethylsilane was used as an internal standard (0 ppm) for ^1^H and ^13^C{^1^H} NMR spectra. Gel permeation chromatography (GPC) measurements were carried out on a SHIMADZU prominence GPC system (Japan) equipped with polystyrene gel columns, using CHCl3 as an eluent after calibration with polystyrene standards. Elemental analyses were carried out using an elementar UNICUBE. Intensity data for crystal structure determination were collected on a Rigaku XtaLAB Synergy-R with Cu Kα radiation. The crystals used for the analysis were prepared from a concentrated hexane solution of **(*R*)-TPD**. The crystal was mounted using MicroMounts (MiTeGen). Crystallographic data for **(*R*)-TPD** have been deposited with the Cambridge Crystallographic Data Centre as supplementary publication CCDC 2,533,781. UV–Vis absorption spectra were recorded on a Hitachi U-3900 H (Japan) with a scan speed of 300 nm min^−1^ and a slit width of 2 nm or JASCO V630 spectrophotometer (Japan) with a scan speed of 400 nm min^−1^ and a slit width of 1.5 nm. Photoluminescence spectra were recorded on a Hitachi F-2700 fluorescence spectrophotometer (Japan) with an excitation slit width of 10.0 nm, an emission slit width of 10.0 nm, a photomultiplier tube voltage of 400 V, a response time of 0.08 s, and a scan speed of 1500 nm min^−1^. The PL quantum yields (PLQY) were measured using a Hamamatsu Photonics C9920-02 absolute PL quantum yield spectrometer (Japan). CD measurements were carried out by JASCO J820 (Japan) with a sensitivity set to low, a scan speed of 50 nm min^−1^, a response time of 1 s, and a bandwidth of 1 nm. Measurements were performed in a continuous scanning mode at 25°C with stirring. CPL measurements were carried out by JASCO CPL-300 (Japan) with an excitation wavelength of 400 nm, an excitation bandwidth of 10.0 nm, and an emission bandwidth of 16.0 nm. Measurements were performed in continuous scanning mode at a scan speed of 100 nm min^−1^, with a photomultiplier tube voltage of 700 V, at 25°C under stirring at 600 rpm, with five accumulations. The CD and CPL properties of the polymers were evaluated in solution at a repeating-unit concentration of 3.0 × 10^−5^ M. An X-ray diffraction pattern was recorded at 298 K on a Rigaku model MultiFlex X-ray diffractometer (Japan) with a CuKα radiation source. The thermal properties were measured on an Seiko Instruments Inc. EXSTAR7000 DSC instrument (Japan). MALDI-TOF-MS spectra were recorded on a Shimadzu AXIMA-CFR Plus (Japan). Fluorescence microscopy observations were carried out using an Olympus model BX53 upright microscope (Japan). Scanning electron microscopy (SEM) was performed on a Hitachi Model S-3700N SEM (Japan) operating at 15 kV. Dry solvents were purchased from Kanto Chemical. Pd(OAc)_2_ (Kojima Chemicals Co., Ltd.) was used as a catalyst. The other reagents were purchased from Tokyo Chemical Industry Co., Ltd. (TCI), Sigma-Aldrich and FUJIFILM Wako Pure Chemical Corporation. DFT calculations were performed at the B3LYP/6-31 G(d) level using Gaussian 16 package.

### Synthesis

#### Synthesis of (R)-5-(1-cyclohexylethyl)-4H-thieno[3,4-c]pyrrole-4,6(5H)-dione, ((R)-TPD)

A 25 mL Schlenk tube charged with a magnetic stir bar was loaded with 3,4-thiophenedicarboxylic acid (172.2 mg, 1.0 mmol) and acetic anhydride (3.23 mL), and the mixture was refluxed at 140°C for 6 h. After cooling to room temperature, the reaction mixture was dried under vacuum. toluene (3.23 mL) and (*R*)-1-cyclohexylethylamine (219 μL, 1.5 mmol) were added, and the mixture was refluxed at 120°C for 24 h under a nitrogen atmosphere. After cooling to room temperature, the mixture was dried under vacuum. Thionyl chloride (1.1 mL) was then added, and the mixture was refluxed at 72°C for 3 h under a nitrogen atmosphere. After cooling to room temperature, the reaction mixture was dried under vacuum. The resulting solids in the Schlenk tube were extracted with *n*-hexane, washed three times with distilled water and once with saturated brine. The organic layer was dried over Na_2_SO_4_ and concentrated under reduced pressure. The crude product was purified by silica gel column chromatography (eluent: CHCl_3_/*n*-hexane = 60:40), followed by HPLC. After vacuum drying at 60°C for 2 h, ***(R)*****-TPD** was obtained as a white solid (181.9 mg, 69% yield).

^1^H NMR (600 MHz, CDCl_3_): δ = 7.78 (s, 2 H), 3.93 (dd, *J* = 10.2, 7.2 Hz, 1 H), 2.02 (q, *J* = 11.4 Hz, 1 H), 1.90 (d, *J* = 13.2 Hz, 1 H), 1.76 (d, *J* = 13.8 Hz, 1 H), 1.64 (t, *J* = 10.8 Hz, 2 H), 1.56 (d, *J* = 13.8 Hz, 1 H), 1.44 (d, *J* = 6.0 Hz, 3 H), 1.27 (dd, *J* = 14.4, 12.0 Hz, 1 H), 1.21–1.09 (m, 2 H), 0.98–0.86 (m, 2 H). ^13^C{^1^H} NMR (600 MHz, CDCl_3_): δ = 162.88, 136.68, 125.22, 52.92, 39.82, 30.59, 30.19, 26.20, 25.87, 25.77, 16.24. EA: Found. C 63.68%, H 6.53%, N 5.25%, S 12.29%; Calcd. for C_14_H_17_NO_2_S: C 63.85%, H 6.51%, N 5.32%, S 12.17%. APCI-TOF-MS: *m/z* Calcd. For C_14_H_18_NO_2_S ([M+H]) 264.1053, Found 264.1054 (Positive ion mode).

***(S)*****-TPD** was synthesized in an analogous manner, affording the desired product as a white solid (160.1 mg, 61% yield). EA: Found. C 63.58%, H 6.51%, N 5.24%, S 12.24%; Calcd. for C_14_H_17_NO_2_S: C 63.85%, H 6.51%, N 5.32%, S12.17%. APCI-TOF-MS: *m/z* Calcd. For C_14_H_18_NO_2_S ([M+H]) 264.1053, Found 264.1027 (Positive ion mode).

#### Synthesis of (R)-PFTPD

A 25 mL Schlenk tube equipped with a magnetic stir bar was charged with **(*R*)-TPD** (52.67 mg, 0.20 mmol), 2,7-dibromo-9,9-di-*n*-octylfluorene (109.70 mg, 0.20 mmol), and Cs_2_CO_3_ (165.33 mg, 0.51 mmol). Under a nitrogen atmosphere, a toluene solution of Pd(PCy_3_)_2_ (2.67 mg/mL) was added (1 mL, 4.0 μmol), followed by pivalic acid (6.9 μL, 0.060 mmol). The mixture was stirred at 100°C for 24 h. After cooling to room temperature, the reaction mixture was dried under vacuum. The crude mixture was extracted with CHCl_3_, and an aqueous solution of NaS_2_CN(C_2_H_5_)_2_ (1 mol%) was added (150 mL) and stirred overnight. The mixture was washed three times with distilled water and once with saturated brine. The organic layer was dried over Na_2_SO_4_ and concentrated under reduced pressure. The residue was subjected to reprecipitation from methanol and stirred overnight. The resulting solids were collected, washed with *n*-hexane, and dried under vacuum at 60°C for 2 h to afford **(*R*)-PFTPD** as a yellow solid (122.6 mg, 94% yield).

^1^H NMR (600 MHz, CDCl_3_): δ = 8.29–8.18 (m, 4 H), 7.86 (d, *J* =8.4 Hz, 2 H), 4.10 (br, 1 H), 2.17 (br, 4 H), 1.98 (br, 1 H), 1.80 (br, 1 H), 1.69 (br, 3 H), 1.37–0.73 (br, 39 H). ^13^C{^1^H} NMR (600 MHz, CDCl_3_): δ = 163.22, 152.40, 145.13, 142.20, 130.55, 130.21, 127.62, 122.94, 120.62, 55.89, 53.01, 40.11, 39.85, 31.84, 30.76, 30.37, 30.04, 29.25, 29.21, 26.29, 25.98, 25.84, 24.00, 22.63, 16.38, 14.05. *M*_n_ = 45000, *M*_w_/*M*_n_ = 2.1.

**(*S*)-PFTPD** was synthesized in an analogous manner, affording the desired product as a yellow solid (119.1 mg, 92% yield). *M*_n_ = 56000, *M*_w_/*M*_n_ = 2.2.

## Supplementary Material

Supplemental Material

Supplemental Material

Supplemental Material

## Data Availability

The authors confirm that the data supporting the findings of this study are available within the article and its supplementary materials.
