# Supplementary material for: Reproducible chiroptical activity from aggregated chiral thienopyrroledione–fluorene π‑conjugated polymers
Source: Sci Technol Adv Mater. 2026 Jun 2;27(1):2680968. doi: 10.1080/14686996.2026.2680968 (PMC13292314; doi:10.1080/14686996.2026.2680968)

checkCIF/PLATON report

Structure factors have been supplied for datablock(s) exp_139_auto

THIS REPORT IS FOR GUIDANCE ONLY. IF USED AS PART OF A REVIEW PROCEDURE FOR PUBLICATION, IT SHOULD NOT REPLACE THE EXPERTISE OF AN EXPERIENCED CRYSTALLOGRAPHIC REFEREE.

No syntax errors found. CIF dictionary Interpreting this report

Datablock: exp_139_auto

Bond precision: C-C = 0.0037 A Wavelength=1.54184

Cell:	a=11.1019(2)	b=10.5201(1)	c=12.1698(2)
	alpha=90	beta=111.518(2)	gamma=90
Temperature:	100 K		

	Calculated	Reported
Volume	1322.28(4)	1322.28(4)
Space group	P 21	P 1 21 1
Hall group	P 2yb	P 2yb
Moiety formula	C14 H17 N O2 S, C12.05 H13.42 N O2 S, C1.95 H3.59	2(C14 H17 N O2 S)
Sum formula	C28 H34 N2 O4 S2	C28 H34 N2 O4 S2
Mr	526.69	526.69
Dx,g cm-3	1.323	1.323
Z	2	2
Mu (mm-1)	2.124	2.124
F000	560.0	560.0
F000'	562.80	
h,k,lmax	14,13,15	13,13,15
Nref	5568[2941]	5334
Tmin,Tmax	0.775,0.809	0.655,1.000
Tmin'	0.654	

Correction method= # Reported T Limits: Tmin=0.655 Tmax=1.000
AbsCorr = MULTI-SCAN

Data completeness= 1.81/0.96 Theta(max)= 76.592

R(reflections)= 0.0323(5259) wR2(reflections)= 0.0821(5334)

S = 1.018 Npar= 327

The following ALERTS were generated. Each ALERT has the format
test-name_ALERT_alert-type_alert-level.
Click on the hyperlinks for more details of the test.

Alert level C

PLAT042_ALERT_1_C Calc. and Reported MoietyFormula Strings Differ Please Check
Calc: C14 H17 N O2 S, C12.05 H13.42 N O2 S, C1.95 H3 .59
Rep.: 2(C14 H17 N O2 S)
PLAT202_ALERT_3_C Isotropic non-H Atoms in Anion/Solvent .... 6 Check
C28 C30 C31 C32 C33 C34
PLAT250_ALERT_2_C Large U3/U1 Ratio for <U(i,j)> Tensor(Resd 2) 2.1 Note
PLAT329_ALERT_4_C Carbon Atom Hybridisation Unclear for .... C32A Check

---

Alert level G

PLAT002_ALERT_2_G Number of Distance or Angle Restraints on AtSite 3 Note
PLAT142_ALERT_4_G s.u. on b - Axis Small or Missing .... 0.00010 Ang.
PLAT176_ALERT_4_G The CIF-Embedded .res File Contains SADI Records 14 Report
PLAT302_ALERT_4_G Anion/Solvent/Minor-Residue Disorder (Resd 2) 25% Note
PLAT302_ALERT_4_G Anion/Solvent/Minor-Residue Disorder (Resd 3) 100% Note
PLAT304_ALERT_4_G Non-Integer Number of Atoms in ..... (Resd 2) 29.46 Check
PLAT304_ALERT_4_G Non-Integer Number of Atoms in ..... (Resd 3) 5.54 Check
PLAT412_ALERT_2_G Short Intra XH3 .. XHn H22B ..H33A . 2.11 Ang.
x,y,z = 1_555 Check
PLAT432_ALERT_2_G Short Inter X...Y Contact N2 ..C32A . 2.67 Ang.
x,y,z = 1_555 Check
PLAT432_ALERT_2_G Short Inter X...Y Contact C20 ..C32A . 3.14 Ang.
x,y,z = 1_555 Check
PLAT432_ALERT_2_G Short Inter X...Y Contact C21 ..C32A . 1.80 Ang.
x,y,z = 1_555 Check
PLAT432_ALERT_2_G Short Inter X...Y Contact C21 ..C33A . 2.74 Ang.
x,y,z = 1_555 Check
PLAT432_ALERT_2_G Short Inter X...Y Contact C21 ..C28A . 2.85 Ang.
x,y,z = 1_555 Check
PLAT432_ALERT_2_G Short Inter X...Y Contact C22 ..C32A . 2.64 Ang.
x,y,z = 1_555 Check
PLAT773_ALERT_2_G Check long C-C Bond in CIF: C21 --C32A 1.80 Ang.
PLAT791_ALERT_4_G Model has Chirality at C7 (Sohncke SpGr) R Verify
PLAT791_ALERT_4_G Model has Chirality at C21 (Sohncke SpGr) R Verify
PLAT860_ALERT_3_G Number of Least-Squares Restraints .... 2 Note
PLAT912_ALERT_4_G Missing # of FCF Reflections Above STh/L= 0.600 52 Note
PLAT969_ALERT_5_G The 'Henn et al.' R-Factor-gap value .... 3.674 Note
Predicted wR2: Based on SigI**2 2.23 or SHELX Weight 8.06
PLAT978_ALERT_2_G Number C-C Bonds with Positive Residual Density. 13 Info
PLAT994_ALERT_1_G SHELXL .ins Contains no or MERG 0 Instruction .. ! Note

```
 0 ALERT level A = Most likely a serious problem - resolve or explain
 0 ALERT level B = A potentially serious problem, consider carefully
 4 ALERT level C = Check. Ensure it is not caused by an omission or oversight
22 ALERT level G = General information/check it is not something unexpected

 2 ALERT type 1 CIF construction/syntax error, inconsistent or missing data
11 ALERT type 2 Indicator that the structure model may be wrong or deficient
 2 ALERT type 3 Indicator that the structure quality may be low
10 ALERT type 4 Improvement, methodology, query or suggestion
 1 ALERT type 5 Informative message, check
```

It is advisable to attempt to resolve as many as possible of the alerts in all categories. Often the minor alerts point to easily fixed oversights, errors and omissions in your CIF or refinement strategy, so attention to these fine details can be worthwhile. It is up to the individual to critically assess their own results and, if necessary, seek expert advice.

PLATON version of 15/01/2026; check.def file version of 02/01/2026

Datablock exp_139_auto - ellipsoid plot

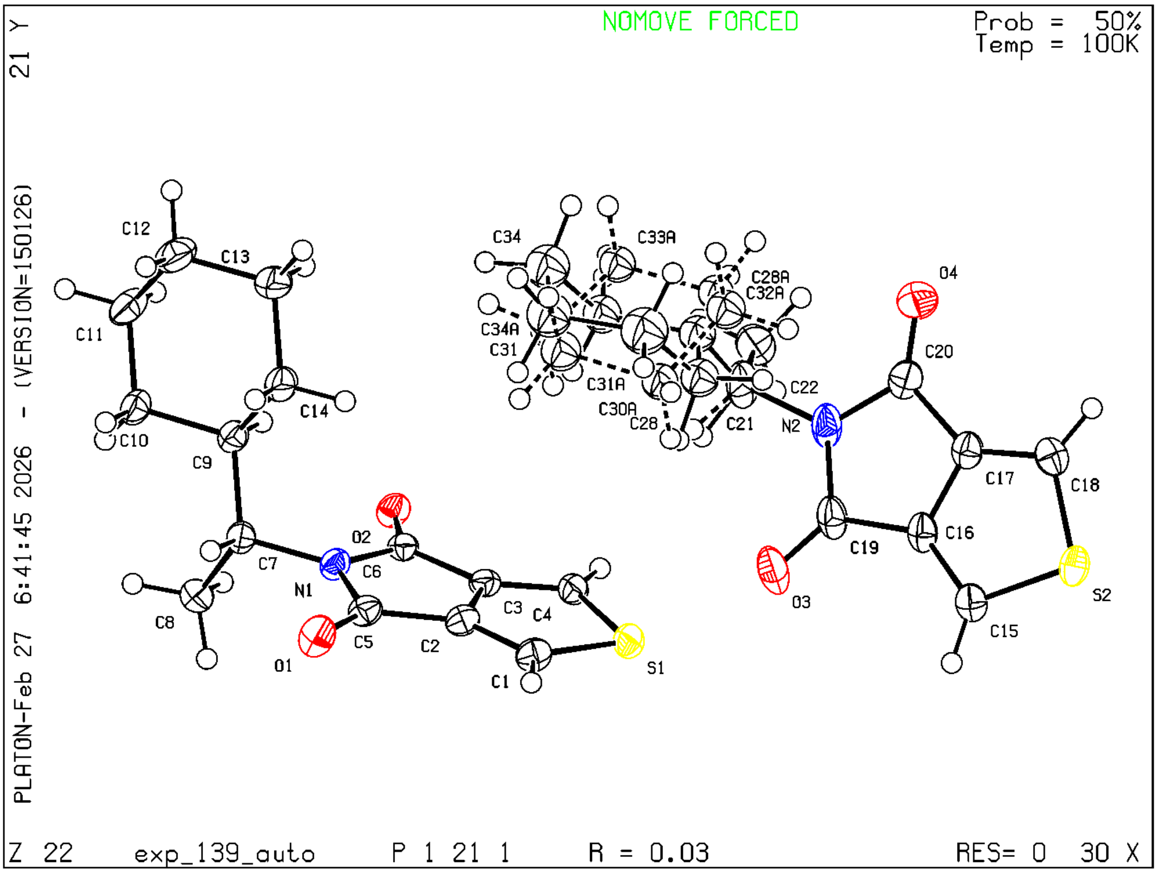

Supplement: Supplemental Material [file TSTA_A_2680968_SM3424.pdf]
