# Supplementary material for: Reproducible chiroptical activity from aggregated chiral thienopyrroledione–fluorene π‑conjugated polymers
Source: Sci Technol Adv Mater. 2026 Jun 2;27(1):2680968. doi: 10.1080/14686996.2026.2680968 (PMC13292314; doi:10.1080/14686996.2026.2680968)
Supplement: Supplemental Material [file TSTA_A_2680968_SM3423.docx]

Supporting information

Reproducible chiroptical activity from aggregated chiral thienopyrroledione–fluorene π‑conjugated polymers.

Nao Suzuki,^a^ Ziwei Hu,^a^ Sota Nakayama,^a^ Soh Kushida,^a^ Yohei Yamamoto,^a,b^ Wijak Yospanya,^c^ Reiko Oda,^d,c^ Takaki Kanbara,^a^ Junpei Kuwabara^a,b *^

^a^ Institute of Pure and Applied Sciences, University of Tsukuba 1-1-1 Tennodai, Tsukuba, Ibaraki 305-8573, Japan.

^b^ Tsukuba Research Center for Energy Materials Science (TREMS), Institute of Pure and Applied Sciences, University of Tsukuba, 1-1-1 Tennodai, Tsukuba, Ibaraki 305-8573, Japan.

^c^ Advanced Institute for Materials Research (AIMR), Tohoku University, 2-1-1 Katahira, Aoba, Sendai, Miyagi, Japan

^d^ University of Bordeaux, CNRS, Bordeaux INP, CBMN, UMR 5248, F-33600 Pessac, France

**E-mail:** kuwabara@ims.tsukuba.ac.jp


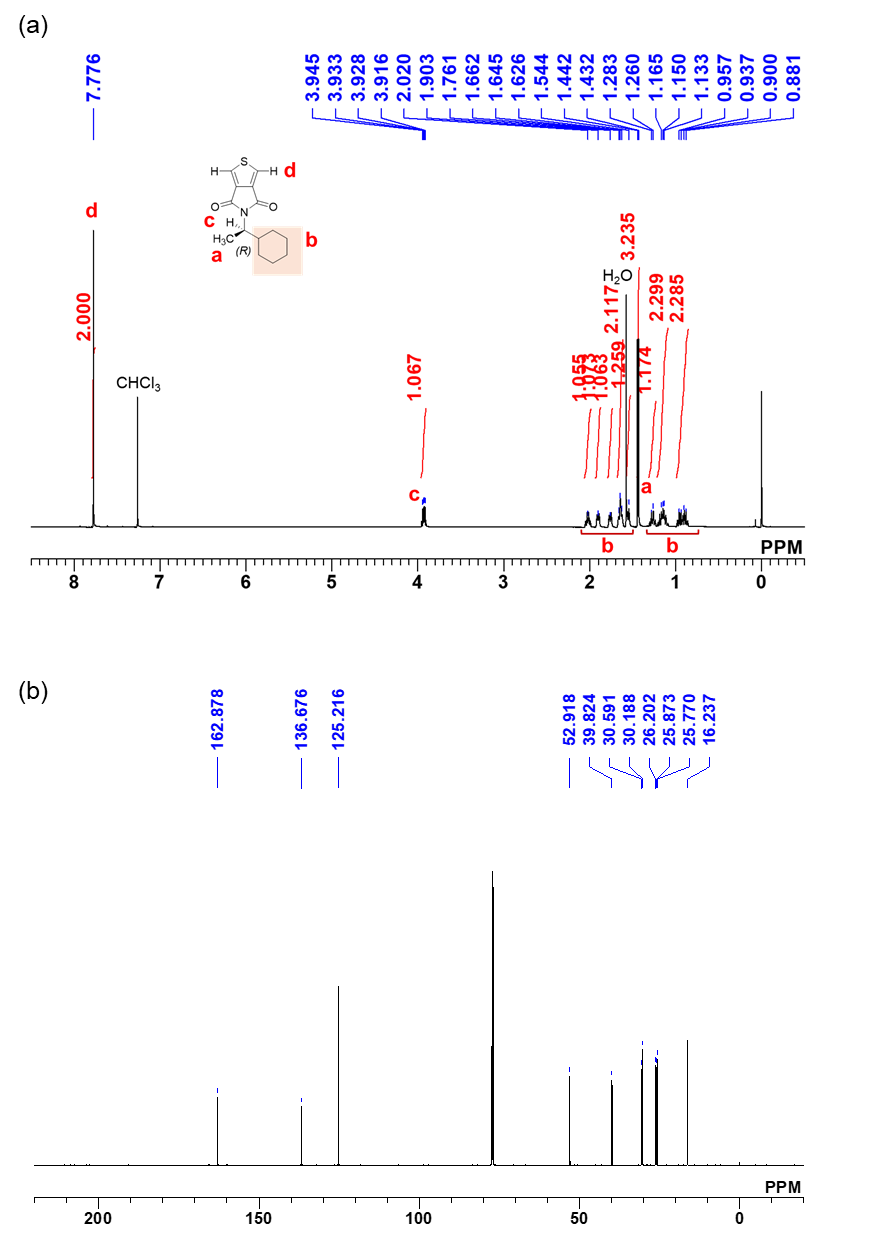


Figure S1. (a) ^1^H NMR (CDCl_3_, 600 MHz, r.t.) and (b) ^13^C{^1^H} NMR spectrum of ***(R)*-TPD** (CDCl_3_, 150 MHz, r.t.).

**
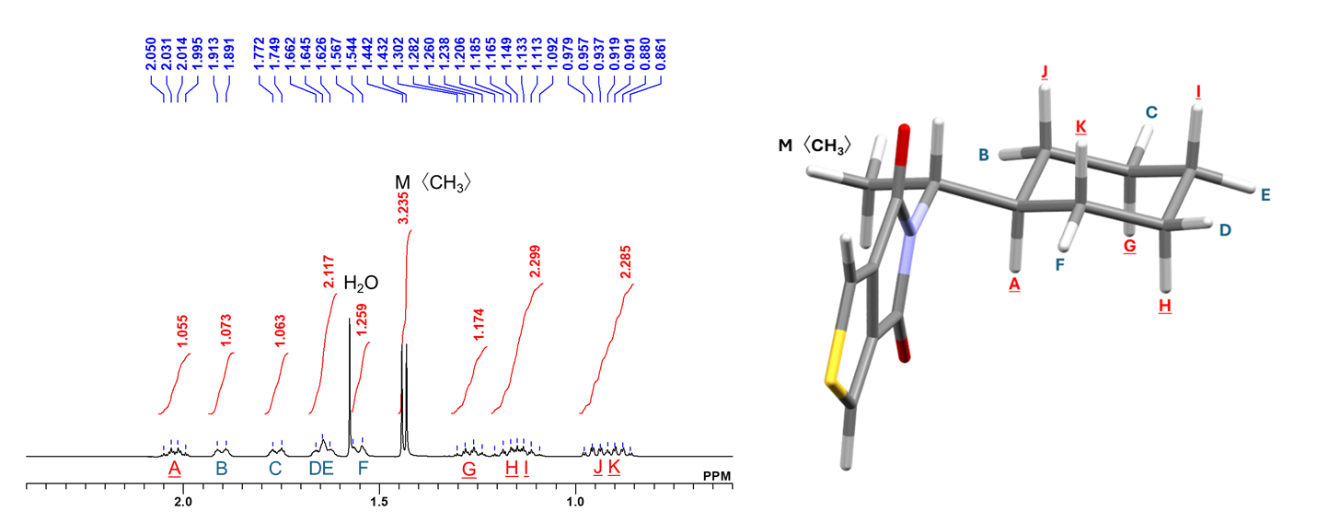
**

Figure S2. ^1^H NMR spectrum of ***(R)*-TPD** (CDCl_3_, 600 MHz, r.t., 0.6-2.4 ppm)

**
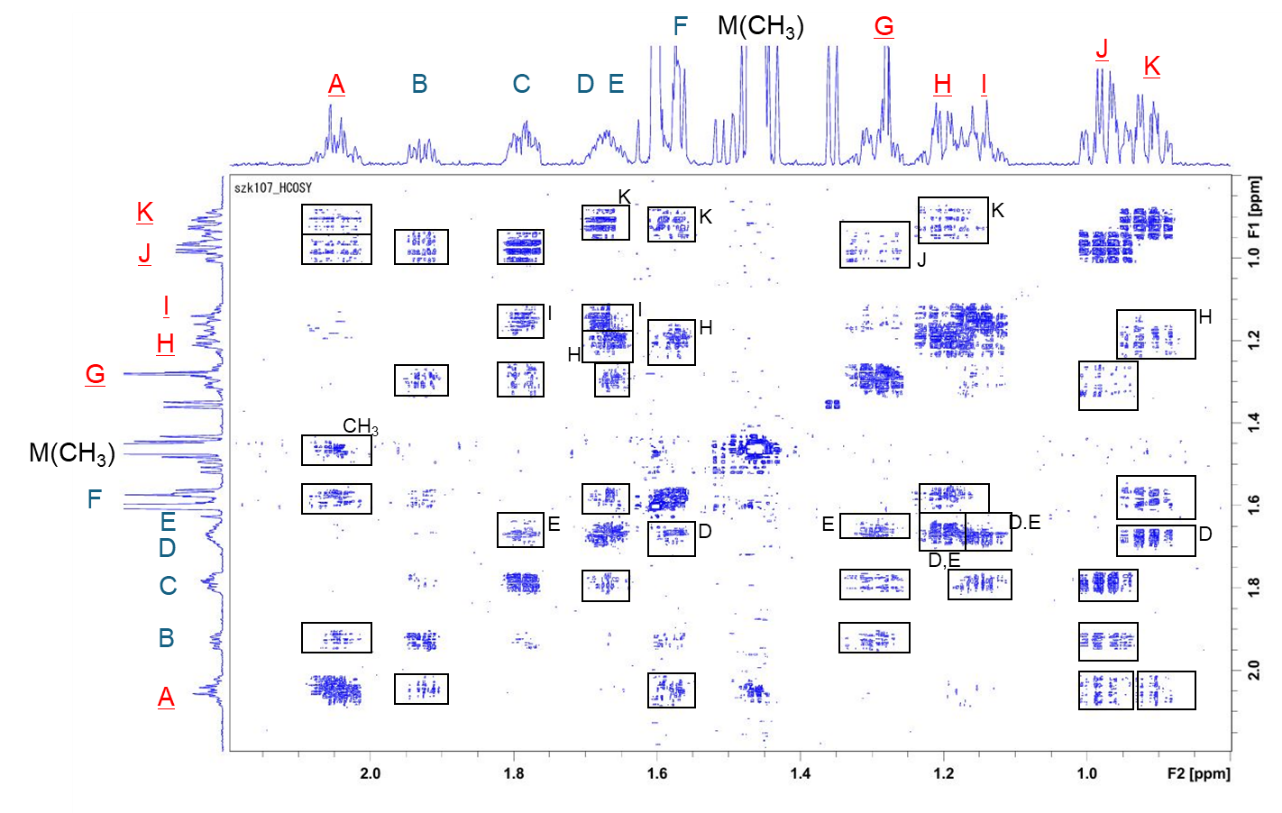
**

Figure S3. ^1^H-^1^H COSY spectrum of ***(R)*-TPD** (CDCl_3_, 600 MHz, r.t., 0.6-2.4 ppm).


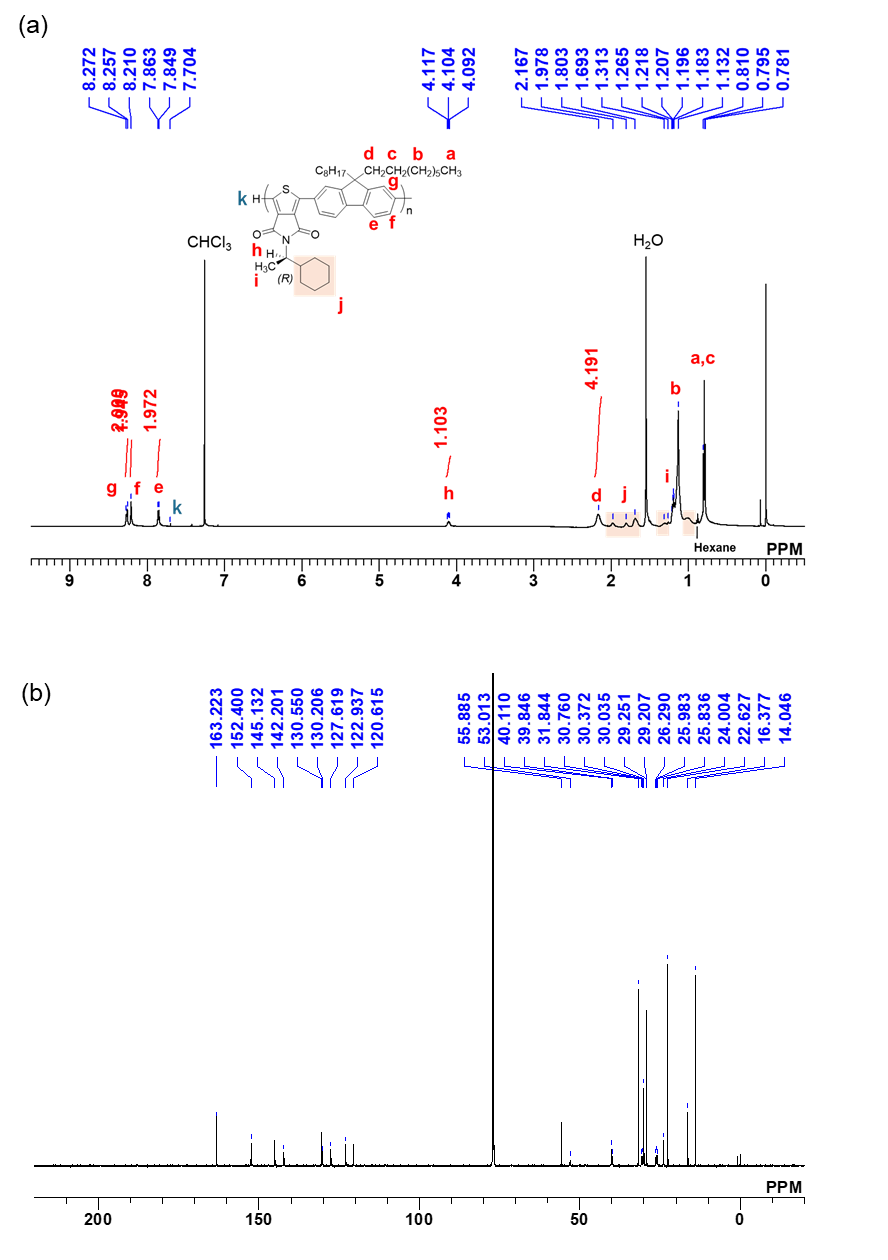


Figure S4. (a) ^1^H NMR (CDCl_3_, 600 MHz, r.t.) and (b) ^13^C{^1^H} NMR spectra of ***(R)*-PFTPD** (CDCl_3_, 150 MHz, r.t.).


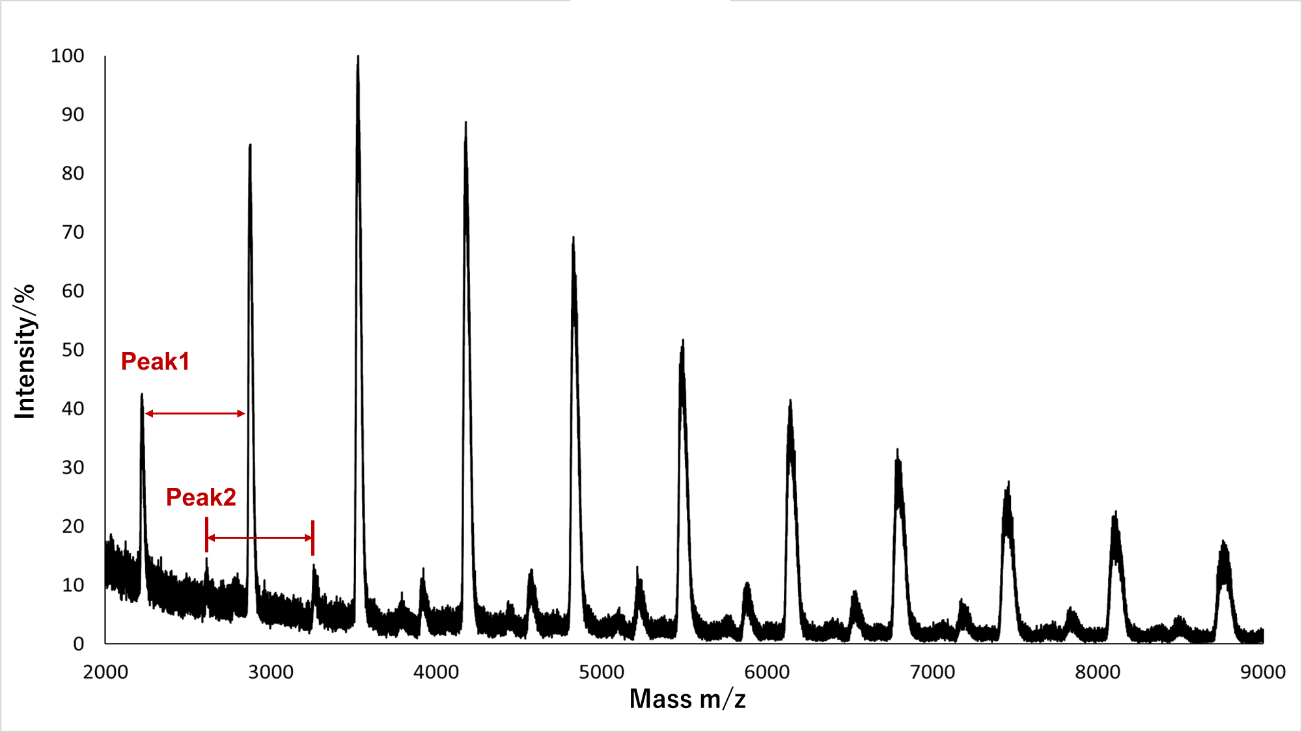


**
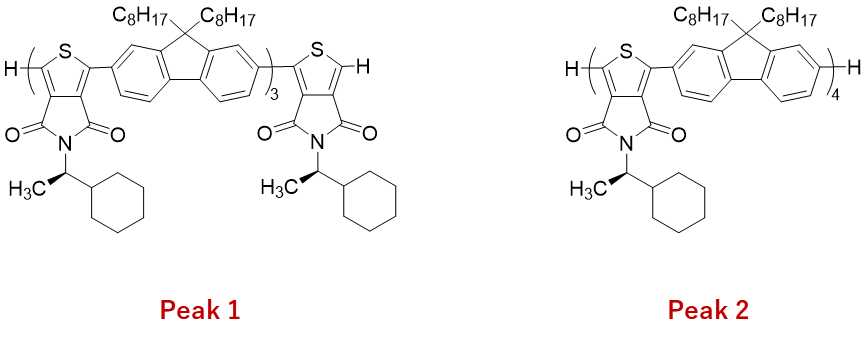
**

Figure S5. MALDI-TOF-MS spectrum of ***(R)*-PFTPD**.


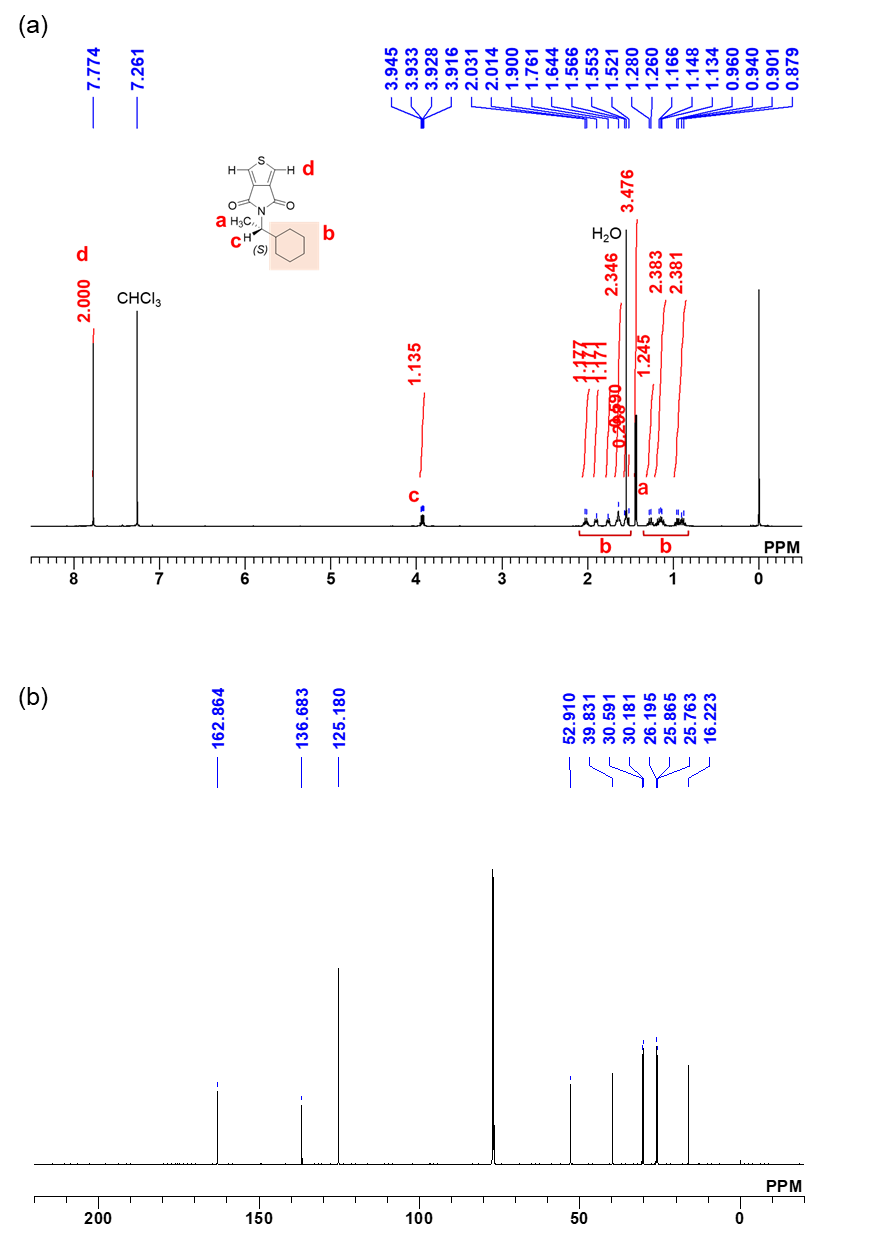


Figure S6. (a) ^1^H NMR (CDCl_3_, 600 MHz, r.t.) and (b) ^13^C{^1^H} NMR spectra of ***(S)*-TPD** (CDCl_3_, 150 MHz, r.t.).


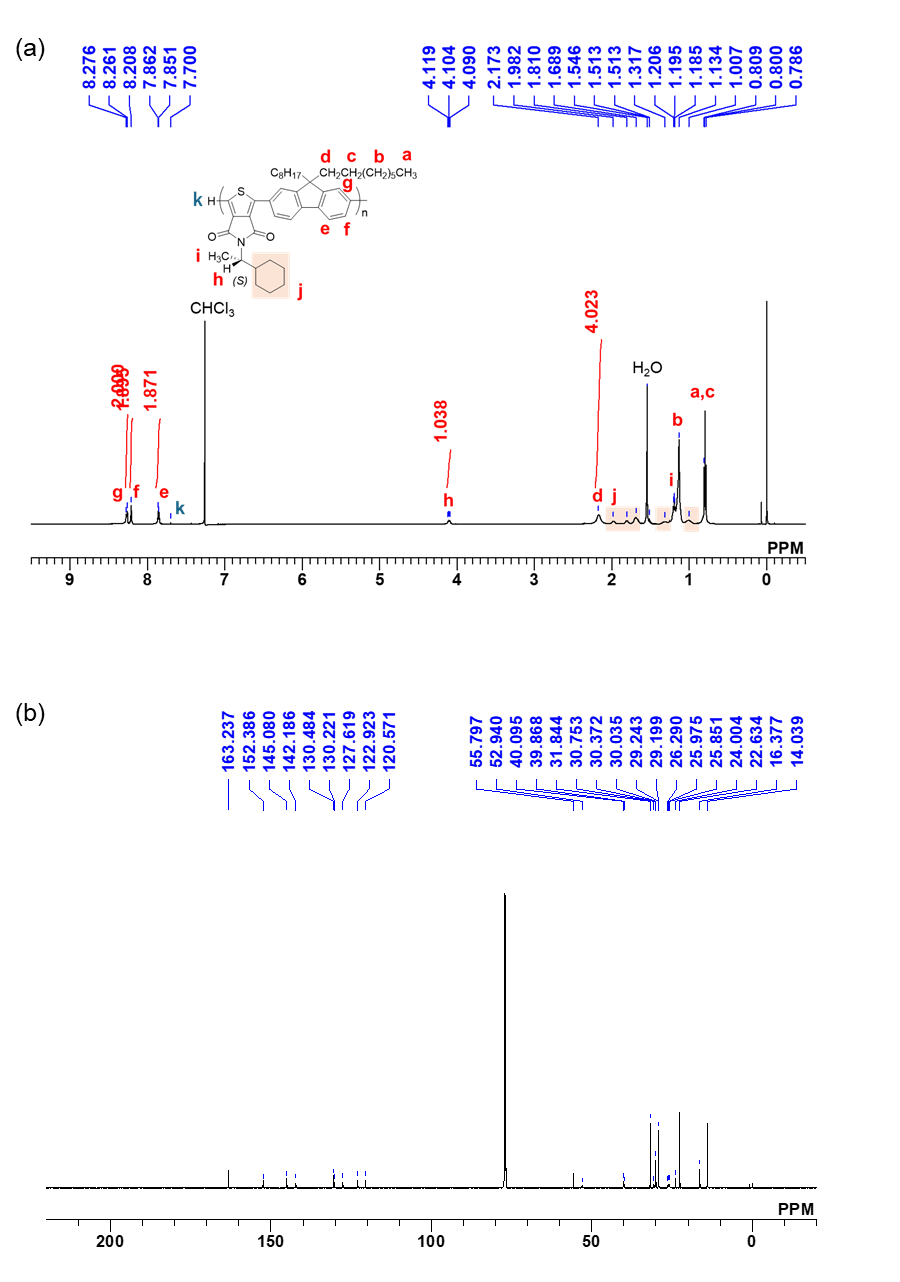


Figure S7. (a) ^1^H NMR (CDCl_3_, 600 MHz, r.t.) and (b) ^13^C{^1^H} NMR spectra of **(*S*)-PFTPD** (CDCl_3_, 150 MHz, r.t.).

**
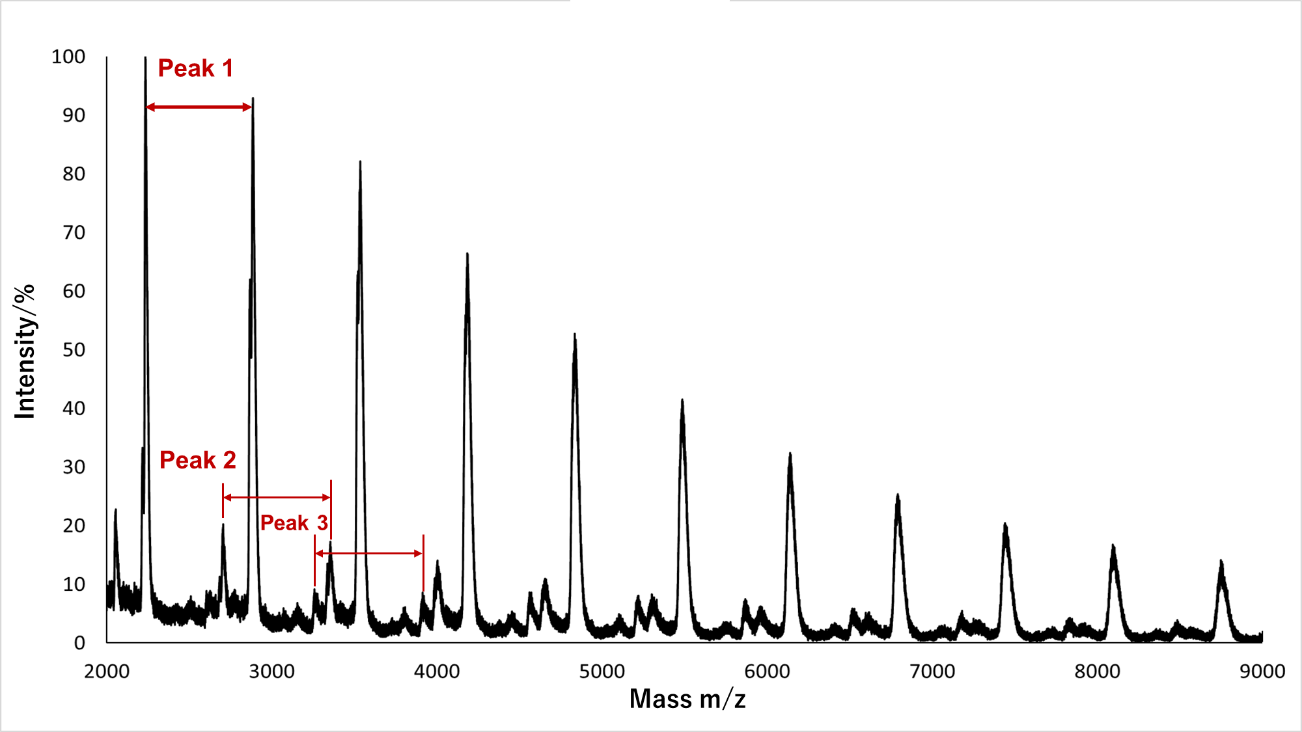
**

**
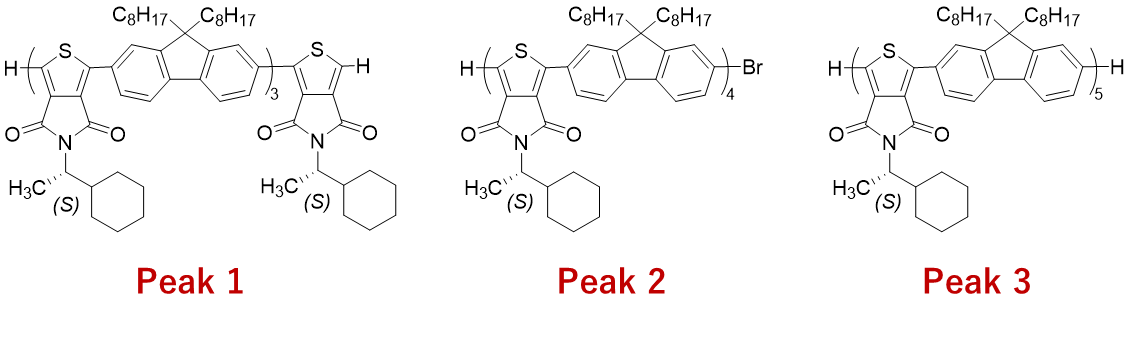
**

Figure S8. MALDI-TOF-MS spectrum of **(*S*)-PFTPD**.


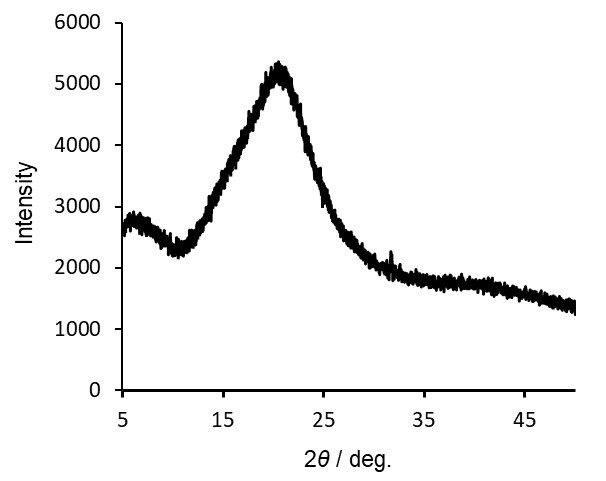


Figure S9. X-ray diffraction (XRD) patterns of a cast film of **(*R*)-PFTPD** measured using Cu*K*α radiation at room temperature.


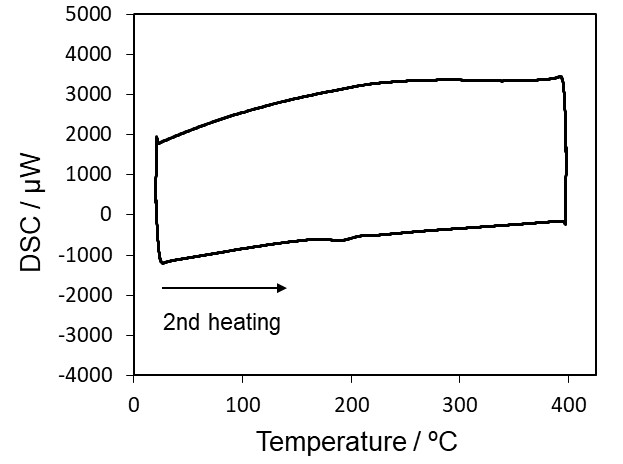


Figure S10. DSC thermograms of **(*R*)-PFTPD** measured under an Ar atmosphere at a heating rate of 10 K min⁻¹ using a sample mass of 4.43 mg. The second heating scan after erasing the thermal history is shown.


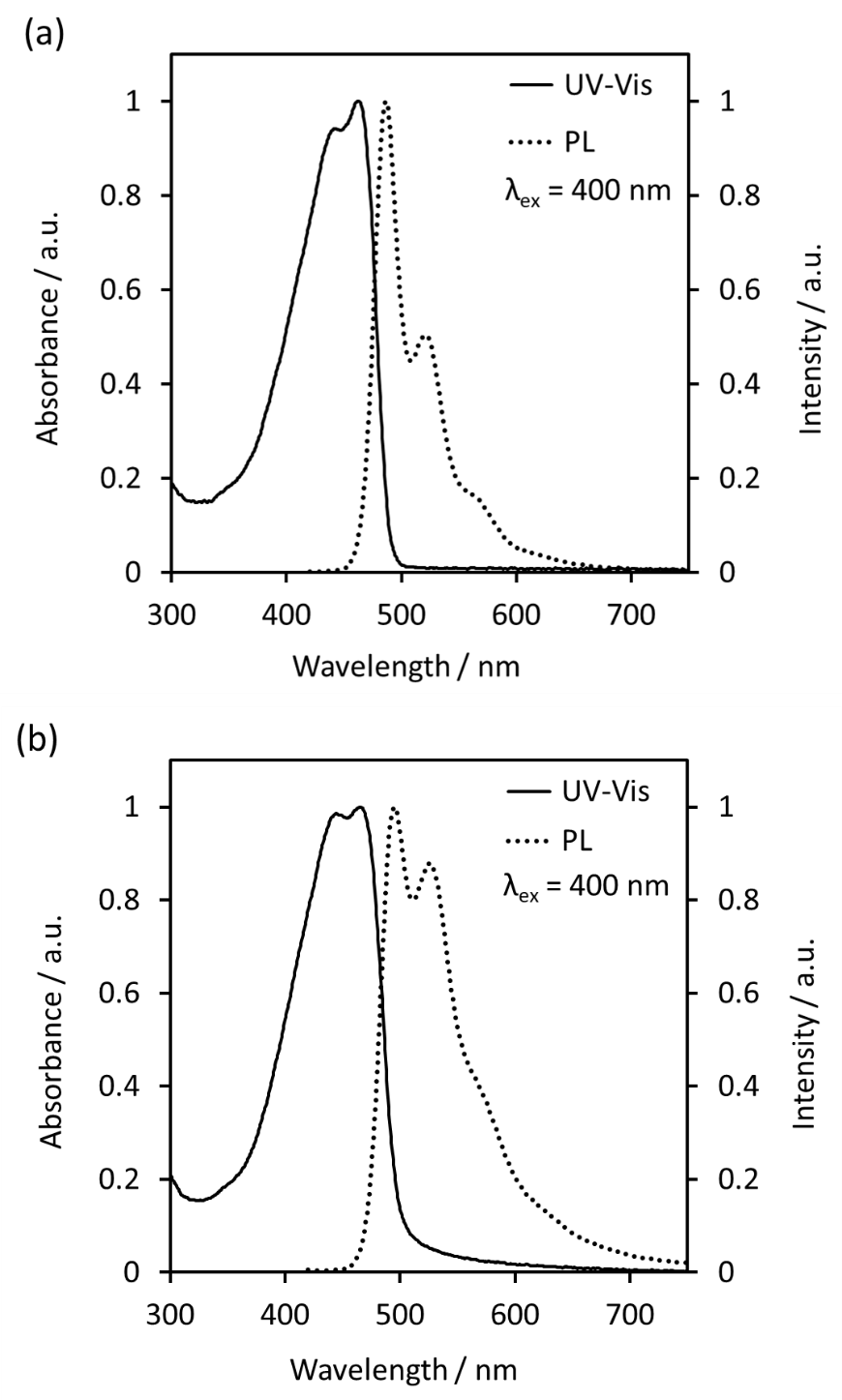


Figure S11. UV-Vis absorption and photoluminescence spectra of **(*R*)-PFTPD** (a) in solution state (CHCl_3_, 5.0 × 10^-6^ M) and (b) in thin film state.


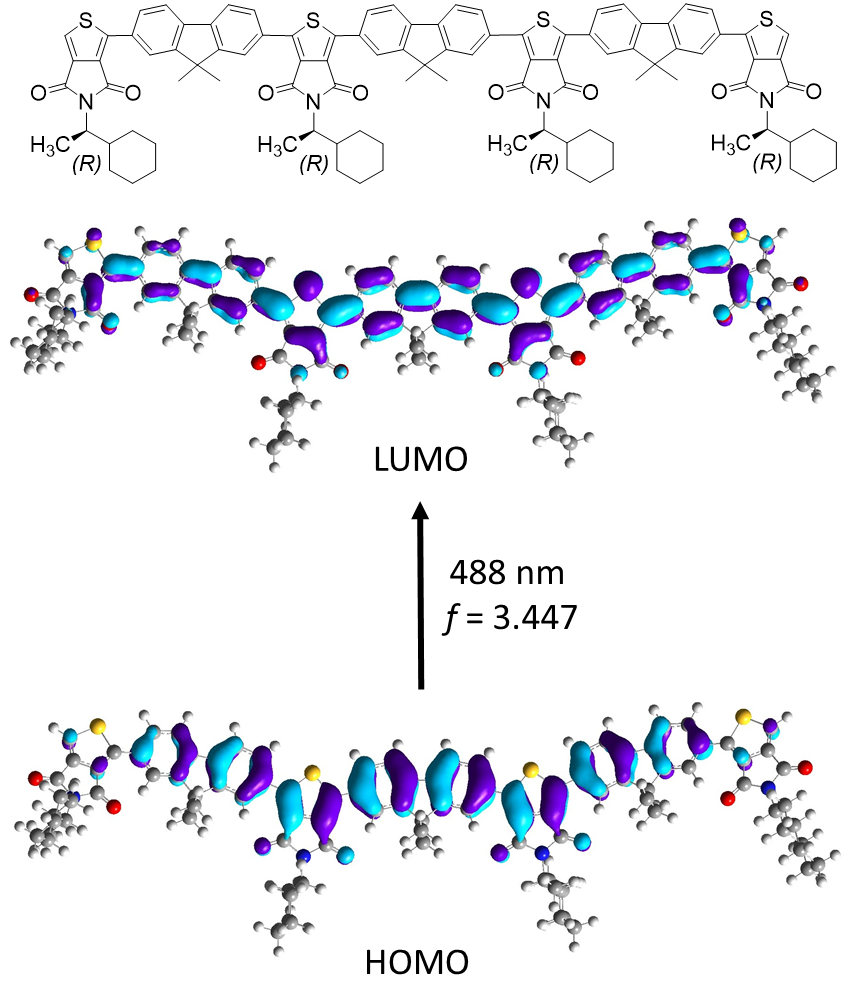


Figure S12. Frontier molecular orbitals (HOMO and LUMO), along with the predicted absorption wavelengths and corresponding oscillator strengths (*f*) obtained from a TD‑DFT calculation.


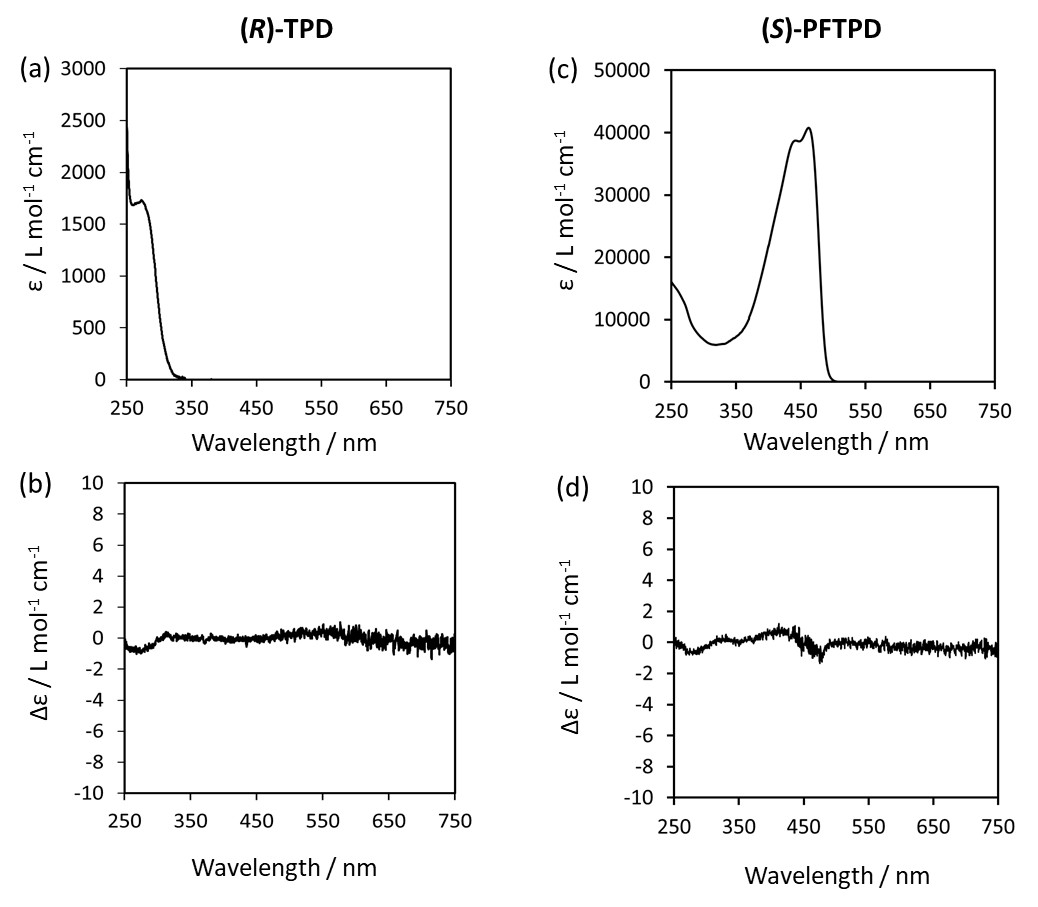


Figure S13. (a) UV–Vis absorption, (b) CD spectra of **(*R*)-TPD**_,_ (c) UV–Vis absorption, and (d) CD spectra of **(*R*)-PFTPD** recorded in CHCl_3_ (3.0 × 10^−5^ M).


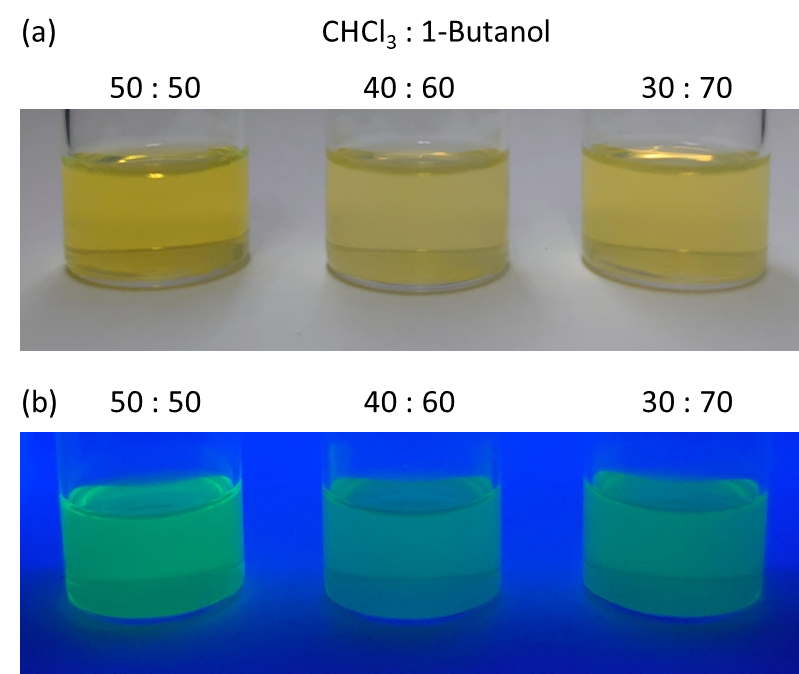


Figure S14. Photographs of **(*R*)-PFTPD** in the different solvent mixtures under natural light and under UV light (365 nm) illumination.


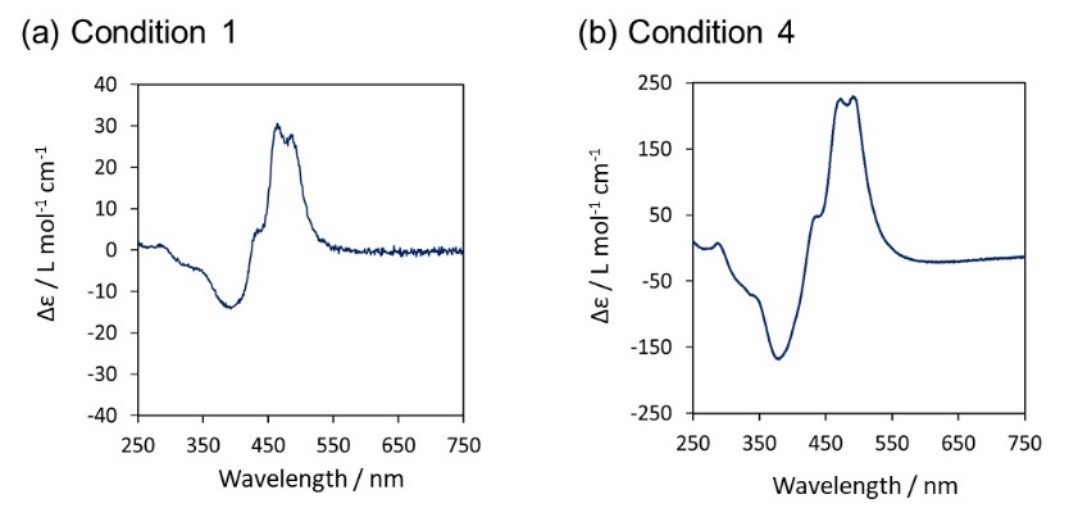


Figure S15. CD spectra of **(*S*)-PFTPD** aggregates prepared under different mixing conditions: (a) Condition 1 and (b) Condition 4. Figure (a) corresponds to the sample listed as Entry 2 in Table S1, while figure (b) corresponds to the sample listed as Entry 30 in Table S1. The spectra were recorded for samples of **(*S*)-PFTPD** in CHCl_3_/1-butanol mixtures with a volume ratio of 40:60 (3.0 × 10^−5^ M).


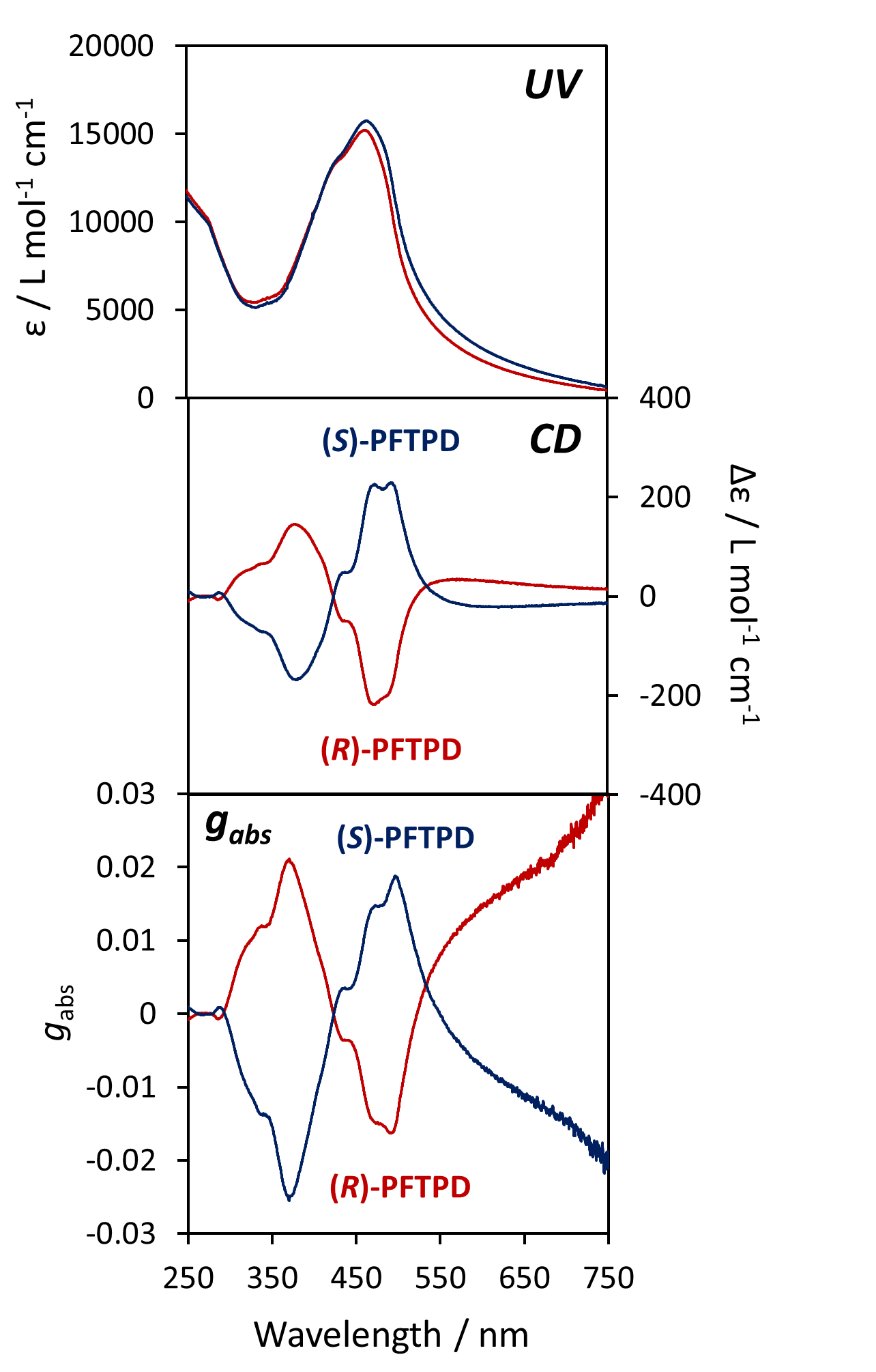


Figure S16. UV-Vis absorption spectra, CD spectra, and *g*_abs_ factors of **(*R*)-PFTPD** (red) and **(S)-PFTPD** (blue) recorded in CHCl_3_/1-butanol mixtures with volume ratios of 40:60 (3.0 × 10^−5^ M).


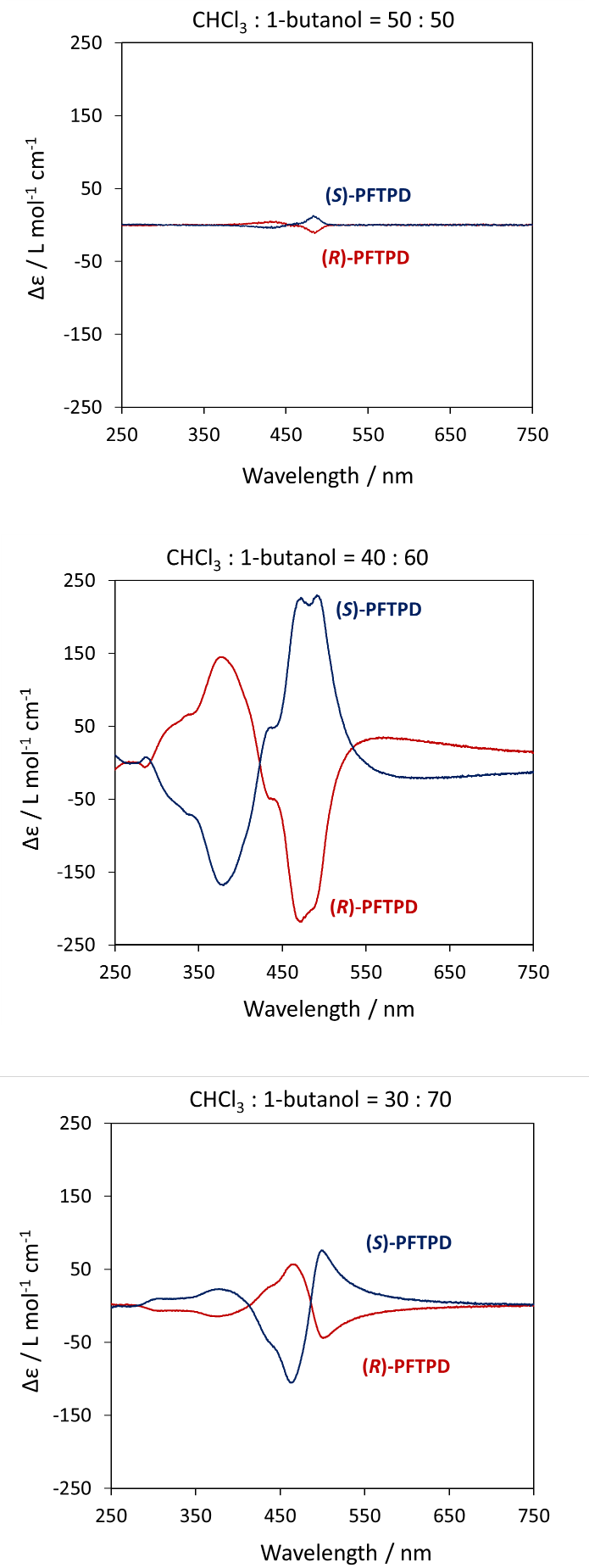


Figure S17. CD spectra of **(*R*)-PFTPD** (red) and **(*S*)-PFTPD** (blue) recorded in CHCl_3_/1-butanol mixtures with volume ratios of 50:50, 40:60, and 30:70 (3.0 × 10^−5^ M).

Table S1. Original *g*_abs_ values corresponding to the preparation conditions. ^a^

| Entry | Condition | *R* or *S* | *g*_abs_ ^f^ |
| --- | --- | --- | --- |
| 1 | 1 ^b^ | *S* | 0.0000874 |
| 2 |  | *S* | 0.00266 |
| 3 | 2 ^c^ | *S* | 0.00595 |
| 4 |  | *S* | 0.00231 |
| 5 |  | *S* | 0.0165 |
| 6 |  | *S* | 0.0128 |
| 7 |  | *S* | 0.00343 |
| 8 |  | *R* | −0.00513 |
| 9 |  | *R* | −0.00349 |
| 10 | 3 ^d^ | *S* | 0.0186 |
| 11 |  | *S* | 0.0173 |
| 12 |  | *S* | 0.0127 |
| 13 |  | *S* | 0.0151 |
| 14 |  | *S* | 0.0277 |
| 15 |  | *S* | 0.0202 |
| 16 |  | *S* | 0.0234 |
| 17 |  | *S* | 0.0158 |
| 18 |  | *S* | 0.00935 |
| 19 |  | *R* | −0.00540 |
| 20 |  | *R* | −0.00770 |
| 21 |  | *R* | −0.0188 |
| 22 |  | *R* | −0.0152 |
| 23 |  | *R* | −0.0127 |
| 24 |  | *R* | −0.0100 |
| 25 |  | *R* | −0.0118 |
| 26 | 4 ^e^ | *S* | 0.0114 |
| 27 |  | *S* | 0.0116 |
| 28 |  | *S* | 0.0132 |
| 29 |  | *S* | 0.0142 |
| 30 |  | *S* | 0.0181 |
| 31 |  | *R* | −0.0150 |
| 32 |  | *R* | −0.0190 |
| 33 |  | *R* | −0.0153 |
| 34 |  | *R* | −0.0175 |

^a^ 1‑BuOH (6 mL) was added to a 75 μM solution of **(*R*)-PFTPD** or **(*S*)-PFTPD** in CHCl_3_ (4 mL). The conditions are shown in Table 2.

^b^ 1‑Butanol was slowly poured into the polymer solution in CHCl_3_, allowing the two solvents to diffuse gradually into each other.

^c^ 1‑Butanol was added all at once to the polymer solution in CHC_3_, followed by manual shaking to promote immediate mixing.

^d^ 1‑Butanol was added dropwise to the polymer solution in CHCl_3_ under stirring.

^e^ 1‑Butanol was added dropwise to the polymer solution in CHCl_3_ under stirring (450 rpm).

^f^ *g*_abs_ values at the peak wavelengths around 480 nm.

Table S2. Representative *g*_abs_ values of **(*R*)-PFTPD**. ^a^

| Solvent ratio  CHCl_3_ : 1-butanol | *g*_abs_ | Wavelength |
| --- | --- | --- |
| 100 : 0 | ~ 0 | - |
| 50 : 50 | −4.2 × 10^−4^ | 485 |
| 40 : 60 | −1.6 × 10^−2^ | 491 |
| 30 : 70 | −6.8 × 10^−3^ | 513 |

^a^ *g*_abs_ values calculated from the data shown in Figures 3 and S11.

Table S3. Original *g*_lum_ values. ^a^

| *R* or *S* | *g*_lum_ |
| --- | --- |
| *S* | 0.0239 |
| *S* | 0.0190 |
| *S* | 0.0127 |
| *R* | −0.0256 |
| *R* | −0.0168 |
| *R* | −0.0158 |

^a^ *g*_lum_ values **(*R*)-PFTPD** and **(*S*)-PFTPD** recorded in CHCl_3_/1-butanol mixtures with volume ratios of 40:60 (3×10^−5^ M, λ_ex_ = 400 nm).
